# Enhanced Built‐in Electric Field Promotes Photocatalytic Hydrogen Performance of Polymers Derived from the Introduction of B←N Coordination Bond

**DOI:** 10.1002/advs.202204055

**Published:** 2022-10-26

**Authors:** Chenglong Ru, Peiyan Chen, Xuan Wu, Changjuan Chen, Jin Zhang, Hao Zhao, Jincai Wu, Xiaobo Pan

**Affiliations:** ^1^ State Key Laboratory of Applied Organic Chemistry (Lanzhou University) Key Laboratory of Nonferrous Metal Chemistry and Resources Utilization of Gansu Province College of Chemistry and Chemical Engineering Lanzhou University No. 222 South Tianshui Road Lanzhou Gansu 730000 P. R. China; ^2^ School of Physics and Electronic Information Yantai University 30 Qingquan Road Yantai Shandong 264005 China; ^3^ College of Chemistry and Pharmaceutical Engineering Huanghuai University No.76 Kaiyuan Avenue Zhumadian Henan 463000 P. R. China; ^4^ Northwest Institute of Eco‐Environment and Resources Chinese Academy of Sciences Donggang West Road 320 Lanzhou Gansu 730000 P. R. China; ^5^ Key Laboratory of Petroleum Resources Research Chinese Academy of Sciences Donggang West Road 320 Lanzhou Gansu 730000 P. R. China

**Keywords:** B←N coordination bond, built‐in electric field, conjugated polymers, hydrogen, photocatalysts

## Abstract

High concentrations of active carriers on the surface of a semiconductor through energy/electron transfer are the core process in the photocatalytic hydrogen production from water. However, it remains a challenge to significantly improve photocatalytic performance by modifying simple molecular modulation. Herein, a new strategy is proposed to enhance the photocatalytic hydrogen evolution performance using boron and nitrogen elements to construct B←N coordination bonds. Experimental results show that polynaphthopyridine borane (**PNBN**) possessing B←N coordination bonds shows a hydrogen evolution rate of 217.4 µmol h^−1^, which is significantly higher than that of the comparison materials 0 µmol h^−1^ for polyphenylnaphthalene (**PNCC**) and 0.66 µmol h^−1^ for polypyridylnaphthalene (**PNNC**), mainly attributed to the formation of a strong built‐in electric field that promotes the separation of photo‐generated electrons/holes. This work opens up new prospects for the design of highly efficient polymeric photocatalysts at the molecular level.

## Graphical abstract

The introduction of B, N elements successfully constructs B←N coordination bonds, which significantly change the electronic properties of the structure. The dramatic improvement in photocatalytic hydrogen precipitation performance is mainly attributed to the strong built‐in electric field, which promotes the separation of the photo‐generated electrons/holes.

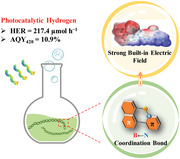

## 1 Introduction

Visible light‐driven hydrogen evolution using semiconductors from water is an important synthesis technology of renewable clean energy with great potential to solve the growing energy and environmental issues worldwide. Photocatalysts are key to the ability to achieve this process. Therefore, the development of efficient photocatalysts has become one of the current research hotspots in the field of photocatalysis. The performance of photocatalysts is influenced by various factors, including light absorption ability, the separation and transport ability of photo‐generated electrons/holes, and the surface catalytic ability, among which the separation and transport ability of photo‐generated electrons/holes are crucial to the influence of the hydrogen evolution performance of photocatalysts.^[^ ^1^, ^2^, ^3^, ^4^, ^5^ ^]^ Recently, conjugated polymer photocatalysts have received much attention as a potential alternative to conventional inorganic semiconductor materials.^[^ ^6^, ^7^, ^8^ ^]^ It has been found that the introduction of heteroatoms into the conjugated backbone of polymers to tune the electronic structure of the materials can effectively improve the separation and transport properties of photo‐generated electrons/holes.^[^ ^9^, ^10^, ^11^, ^12^ ^]^ For example, pyridine,^[^ ^13^, ^14^, ^15^ ^]^ pyrazine,^[^ ^11^, ^16^, ^17^ ^]^ triazine,^[^ ^18^, ^19^, ^20^, ^21^ ^]^ and heptazine,^[^ ^22^, ^23^ ^]^ which contain nitrogen (N)‐heterocycles with C=N double bonds, are often used as effective building blocks for photocatalysts due to their high electron affinity leading to enhanced the transport ability of photo‐generated carriers.^[^ ^24^ ^]^ However, it remains a challenge to significantly alter the separation and transport capacity of photo‐generated carriers by simple molecular modulation and thus achieve a breakthrough in photocatalytic performance.

**Figure 1:**
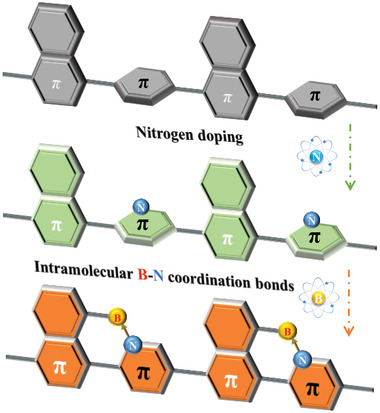
Schematic diagram of electronic and structural modification of the conjugated system by nitrogen doping and B←N coordination.

A common strategy to improve photocatalytic hydrogen production performance is to use alternating electron‐accepting units and electron‐donating units along the backbone to construct donor–accept (D‐A)‐type conjugated polymer photocatalysts.^[^ ^16^, ^25^, ^26^ ^]^ The electronic push–pull ability of the D–A structure to increase charge separation and transport is strongly related to the formation of strong molecular dipoles. However, obtaining polymeric photocatalysts with strong dipoles at the molecular level is still not easy. Recently, our group reported a series of novel conjugated materials with organoboron acceptors and exhibited excellent photocatalytic performance, in which the introduction of organoboron acceptors facilitated the separation and transport of photo‐generated carriers.^[^ ^27^, ^28^, ^29^ ^]^ Accordingly, we envisioned the introduction of organoboron units on the appropriate N‐heterocyclic backbone to construct intramolecular B←N coordination bonds by changing the electronic structure of the N‐heterocyclic ring using the electron‐deficient property of boron atoms (**Figure** **1**). Such B←N polar units can improve the rigidity and planarity of the conjugated system,^[^ ^30^, ^31^, ^32^, ^33^ ^]^ promote the formation of molecular dipoles, and accompany the establishment of a strong internal electric field, thus facilitating the effective separation and transport of carriers.^[^ ^34^, ^35^, ^36^ ^]^

In this paper, three conjugated polymers, the all‐carbon substituted **PNCC** (polyphenylnaphthalene), the N‐substituted **PNNC** (polypyridylnaphthalene), and the BN cointroduced **PNBN** (polynaphthopyridine borane), were successfully prepared and applied to the study of photocatalytic hydrogen production. The experimental results showed that the hydrogen evolution rates (HERs) of the three samples were 0 µmol h^−1^ for **PNCC**, 0.66 µmol h^−1^ for **PNNC**, and 217.4 µmol h^−1^ for **PNBN**, respectively. The greatly improved hydrogen production performance of **PNBN** was mainly due to the introduction of B←N coordination bonds leading to the enhanced structural planarity of the polymers and the increased built‐in electric field, which in turn greatly facilitates the separation and migration efficiency of the photo‐generated carriers. This work provides a new idea for the design of efficient polymer photocatalysts.

## 2 Results and Discussion

**Figure 2:**
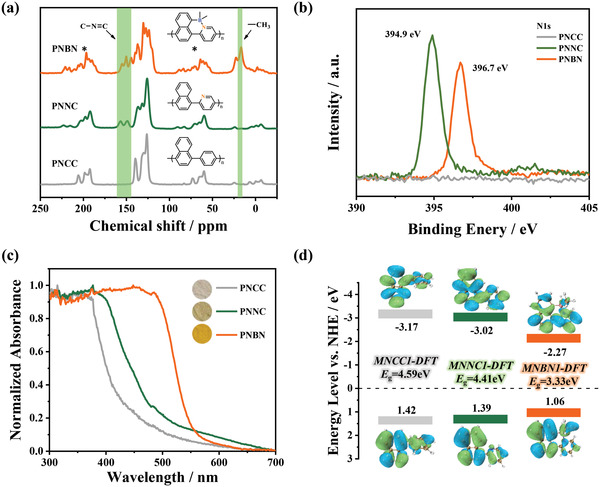
a) Solid‐state ^13^C CP/MAS NMR of polymers **PNCC**, **PNNC**, and **PNBN** (* Represents the sideband). b) XPS N 1s spectra of polymers **PNCC**, **PNNC**, and **PNBN**. c) UV−vis absorption spectra of polymers were measured in the solid state (The embedded images are photos of the polymers). d) HOMO's and LUMO's energy levels and orbits distribution of polymer fragments **MNCC1‐DFT**, **MNNC1‐DFT**, and **MNBN1‐DFT** from DFT calculation.

Polymers **PNCC**, **PNNC**, and **PNBN** were prepared by Pd(0)‐catalyzed Suzuki–Miyaura polymerization in N,N‐dimethylformamide (DMF) in the presence of K_2_CO_3_ at 100 °C for 2 days (Synthesis details are available in the Supporting Information section). The polymers **PNCC**, **PNNC**, and **PNBN** were stable in air and exhibited off‐white, light green, and orange‐yellow apparent colors, respectively. The solid‐state ^13^C cross‐polarization magic angle spinning (^13^C CP/MAS NMR) spectra confirm that the broad signal peaks in the range of 115–145 ppm can be attributed to the aromatic carbon signals of polymer skeleton.^[^ ^37^ ^]^ Moreover, the chemical shifts of **PNNC** at 148 and 156 ppm are attributed to the signal of adjacent carbon on the N atom, which is caused by the lowering of the electron density of the adjacent carbon by the N atom. Due to the lower symmetry, **PNBN** shows multiple complex peaks of aromatic carbon signals in the high frequency region. Meanwhile, the signal at around 20 ppm can be referred to as the characteristic peak of methyl (**Figure** **2**a). In addition, high‐resolution N 1s X‐ray photoelectron spectroscopies (XPS) showed binding energy peaks at 394.9 and 396.7 eV for polymer **PNNC** and **PNBN**, respectively, while sample **PNCC** showed no binding energy peak between 390 and 400 eV, suggesting the presence of pyridine nitrogen in polymers **PNNC** and **PNBN** and the existence of different structural forms (Figure 2b).^[^ ^38^ ^]^ A clear binding energy peak at 184.9 eV was further observed for **PNBN** in the B 1s XPS spectrum, while **PNCC** and **PNNC** had only residual signal peaks for the terminal groups (Bpin and bromine). These results indicate the successful synthesis of N, B‐doped polymer structures (Figure S2, Supporting Information). Furthermore, in the Fourier‐transform infrared (FTIR) spectrum of **PNBN**, the characteristic‐stretching vibrational peak of the C—H bond on the methyl group is located at 2900 cm^−1^ as well as the bending vibrational peak at 1490 cm^−1^, further indicating the formation of the polymeric N, B‐doped structure (Figure S3, Supporting Information).^[^ ^39^, ^40^ ^]^ Powder X‐ray diffraction (PXRD) patterns show (Figure S4, Supporting Information) that the polymers **PNCC** and **PNBN** are semicrystalline, while **PNNC** is amorphous.^[^ ^17^ ^]^ Scanning electron microscopy (SEM) showed that these polymers aggregated into irregular nanoparticles (Figure S5, Supporting Information). The high‐resolution TEM images of **PNCC**, **PNNC**, and **PNBN** were tested and showed that none of the three polymers had significant lattice stripes (Figure S6, Supporting Information). Thus **PNCC**, **PNNC**, and **PNBN**, like most organic polymer materials, do not have ordered crystallinity.

The normalized UV–Vis diffuse reflectance spectra of the samples **PNCC**, **PNNC**, and **PNBN** in the solid state were shown in Figure 2c, where all polymers exhibit intrinsic absorption originating from aromatic *π*–π* electron transitions with maximum absorption wavelengths of 424 (**PNCC**), 468 (**PNNC**), and 547 nm (**PNBN**), respectively. The optical band gaps estimated using the Tauc plot equation were 3.04 eV (**PNCC**), 2.76 eV (**PNNC**), and 2.33 eV (**PNBN**) (Figure S8, Supporting Information), indicating that these polymers have sufficient thermodynamic driving force for the photocatalytic water‐splitting reaction (theoretically 1.23 eV). With the stepwise introduction of N and B atoms, the absorption spectra of samples are significantly red‐shifted and the optical band gap (*E*_g_^opt^) becomes progressively narrower. This trend of evolution may arise for two reasons: first, the formation of B←N coordination bonds fixes the skeleton structure and expands the overall conjugation of the structure;^[^ ^24^, ^32^, ^33^ ^]^ second, the introduction of B←N units alters the donor–acceptor properties of the whole molecule and enhances the intramolecular and intermolecular charge transfer (CT) ability.^[^ ^30^, ^40^, ^41^ ^]^

Density functional theory (DFT) was used to calculate the electronic structure of polymer fragments at the B3LYP/6‐311G (d, p) level (Figure 2d). The highest‐occupied molecular orbital (HOMO) and the lowest‐unoccupied molecular orbital (LUMO) of both **MNCC1‐DFT** (fragment of polyphenylnaphthalene) and **MNNC1‐DFT** (fragment of polypyridylnaphthalene) are uniformly distributed in the aromatic backbone. In contrast, the HOMO and LUMO of **MNBN1‐DFT** (fragment of polynaphthopyridine borane) are located on the naphthyl moiety and pyridyl moieties, respectively. The polarity distribution of the frontier orbitals predicts that the polymer **PNBN** has a distinct D–A characteristic, which facilitates the spatial separation of charges.^[^ ^42^ ^]^ In addition, the HOMO energy of **MNBN1‐DFT** is reduced by 0.36 and 0.33 eV compared with the all‐carbon structure **MNCC1‐DFT** and the nitrogen‐doped **MNNC1‐DFT**, respectively, indicating that the boronized variant **PNBN** has a higher electron affinity.^[^ ^30^, ^43^ ^]^ The calculated results for the longer fragment structure (**MNCC2‐DFT**, **MNNC2‐DFT**, and **MNBN2‐DFT**) show a similar trend (Table S2, Supporting Information). Furthermore, molecular planarity calculations investigated their discrepancies in electron conjugation ability (Figures S10–S13, Supporting Information).^[^ ^44^ ^]^ The introduction of B←N coordination bond limits the rotation of naphthyl and pyridine groups, allowing polymer **PNBN** to exhibit the good planarity with low molecular planarity parameter (MPP = 0.065 Å) and span of deviation from plane (SDP = 0.232 Å). Thus, the intramolecular B←N coordination bond can rigidify a conjugated system consisting of multiple aromatic rings. All results show that the formation of B←N coordination bonds has the potential to promote the photocatalytic hydrogen production performance.

**Figure 3:**
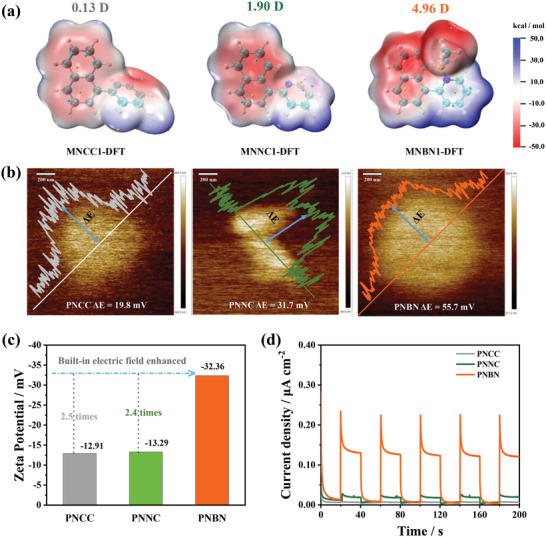
a) Molecular dipole and MESP values mapped on vdW surface of **MNCC1‐DFT**, **MNNC1‐DFT**, and **MNBN1‐DFT**. b) Surface potential of polymers **PNCC**, **PNNC**, and **PNBN** detected with KPFM. c) Zeta potential of polymers **PNCC**, **PNNC**, and **PNBN** in H_2_O. d) Comparison of photocurrent behaviors of polymers **PNCC**, **PNNC**, and **PNBN** under visible light (*λ* > 420 nm, 100 mW cm^−2^).

The molecular electrostatic potential (MESP) on the surface of molecules, van der Waals (vdW) is essential for the study and prediction of intermolecular interactions.^[^ ^45^, ^46^, ^47^ ^]^ As shown in the **Figure** **3**a, electrostatic potential (ESP) of the three model structures differed significantly. **MNNC1‐DFT** and **MNBN1‐DFT** had more significant potential difference compared with the weak electrostatic interaction of **MNCC1‐DFT**. In the ESP area distribution diagram, we find that the percentage of positive charge of **MNBN1‐DFT** increases significantly due to the introduction of boron, with a maximum potential difference of 55.13 eV (Figure S14, Supporting Information). The results of the MESP show that the pyridine group in the backbone of **MNBN1‐DFT** apparently has more electron positivity than the naphthalene group, thus indicating that the B←N coordination enhances the electron‐accepting nature of the pyridine ring. In addition, the molecular dipole moment of the material changes with the doping of N and B. The calculation shows that the dipole of **MNBN1‐DFT** is 4.96 Debye, which is 4.93 and 3.06 Debye higher than the dipoles of **MNCC1‐DFT** (0.13 Debye) and **MNNC1‐DFT** (1.90 Debye), respectively (Figure 3a). The results of the ESP distribution and molecular dipole moment demonstrate at a theoretical level that a large built‐in electric field is formed in the polymer **PNBN**. On the other hand, Zhang et al.^[^ ^48^, ^49^ ^]^ found that the built‐in electric field of the material is positively correlated with the zeta potential and surface potential. The surface potentials of the three materials obtained by Kelvin probe atomic force microscope (AFM) were 19.8 mV for **PNCC**, 31.7 mV for **PNNC**, and 55.7 mV for **PNBN**, respectively (Figure 3b). Obviously, **PNBN** has the largest surface potential. Subsequently, the zeta potential of the polymers in water was tested. As shown in Figure 3c, the zeta potential of **PNBN** (−32.36 mW) was 2.5 times higher than that of **PNCC** (−12.91 mW) and 2.4 times higher than that of **PNNC** (−13.29 mW). In other words, the catalyst **PNBN** shows the strongest built‐in electric field. The enhanced built‐in electric field results in a higher charge separation efficiency on the **PNBN** surface, much higher than that of **PNNC** and **PNCC**. This result is also demonstrated by the photocurrent intensity (Figure 3d) and electrochemical impedance spectroscopy (EIS) (Figure S16, Supporting Information), the photocurrent intensity of the **PNBN** is 0.25 µA cm^−2^, ten times that of the **PNNC** and 30 times higher than that of **PNCC**. The diameter of semicircular Nyquist plots of the **PNBN** at high frequency is smaller than that of **PNCC** and **PNNC**, indicating higher charge separation efficiency and lower charge transfer resistance. These results imply that the polymer **PNBN** possessing B←N coordination bonds is likely to have the best photocatalytic hydrogen evolution activity.

**Figure 4:**
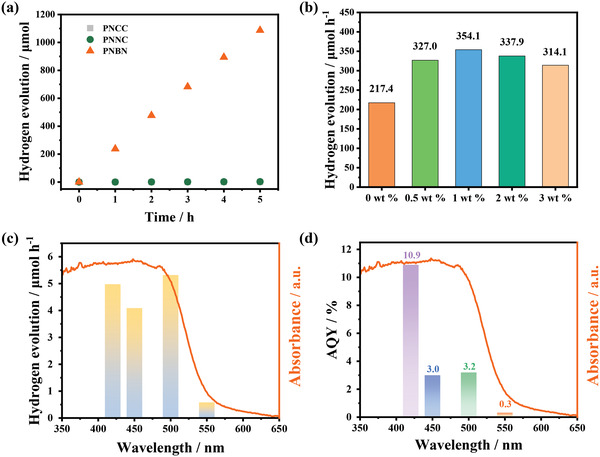
a) Time‐course for photocatalytic hydrogen evolution rate without Pt cocatalysts under visible light irradiation (*λ* > 420 nm, the average result of three experiments). b) HER of **PNBN** with 0, 0.5, 1, 2, and 3 wt% Pt cocatalyst. c) Absorption spectrum and photocatalytic hydrogen evolution rates of **PNBN** at 420, 450, 500, and 550 nm. d) Absorption spectrum and AQYs of **PNBN** at 420, 450, 500, and 550 nm.

The photocatalytic hydrogen production activity of the prepared polymers was evaluated under visible light irradiation (*λ* > 420 nm) in a mixture of water (H_2_O), methanol (CH_3_OH), and triethylamine (TEA) (**Figure** **4**a). Polymer **PNCC** showed almost no hydrogen production under visible light irradiation. In contrast, the HER of the nitrogen‐doped **PNNC** was slightly improved to 0.66 µmol h^−1^, but the photocatalytic activity was still low. Excitingly, the photocatalytic hydrogen evolution activity of the polymer **PNBN** was as high as 217.4 µmol h^−1^ with a turnover number (TON) of 25.74 and a solar‐to‐hydrogen (STH) conversion efficiency (STH) of 1.05% under the same conditions. The HER of **PNBN** increased by several orders of magnitude relative to **PNCC** and **PNNC**, which may be caused by the better planarity and conjugation of the BN heterocyclic structure due to the introduction of B←N coordination bonds. In addition, dynamic light scattering (DLS) experiments (Figure S25, Supporting Information) were performed in methanol. The smaller particle size of **PNBN** can also increase the exposure of catalytic sites on the surface. To confirm the source of H_2_, mass spectrometry of D_2_O was carried out. The prepared catalyst (10.0 mg) was added to 50 mL of a 1:1:1 volume mixture of D_2_O, CH_3_OH, and TEA solution for the photocatalytic reaction. Mass spectrometric analysis (Figure S26, Supporting Information) of the resulting gas showed a deuterium to hydrogen ratio of 0.9633 and a possible source of H as a result of proton exchange between D_2_O and CH_3_OH (the carrier gas in the mass spectrometry test is helium; therefore, only H_2_ and DH can be distinguished.). In addition, the photocatalytic hydrogen evolution rate of **PNBN** remained significant at 142.2 µmol h^−1^ when a mixture of 8 vol% TEA in water was used (Figure S27, Supporting Information). These results indicate that in the system of H_2_O, CH_3_OH, and TEA, the main source of H_2_ is H_2_O as well as the proton exchange of CH_3_OH.

Subsequently, the dependence of the gas evolution rates on the photocatalyst mass, solution pH, and Pt cocatalyst loading was investigated. The results showed a resting point of 0.2 g L^−1^ for **PNBN** (Figure S28, Supporting Information). There was no significant effect on photocatalytic hydrogen evolution in the pH range of 8.8–10 (Figure S29, Supporting Information). In contrast, the loading of the cocatalyst has a significant effect on the photocatalytic activity. The results show a tendency to saturate the performance of HER at lower Pt contents, followed by a decrease at higher Pt concentrations. The optimal HER of **PNBN** reached 354.1 µmol h^−1^ when 1 wt% of Pt was added (Figure 4b). **PNBN** exhibits highly photocatalytic hydrogen production performance compared with the recently reported linear conjugated polymers.^[^ ^50^, ^51^, ^52^, ^53^ ^]^ In addition, the wavelength‐dependent HER and apparent quantum yield (AQY) of the polymer **PNBN** were tested. The results show that the HER is positively correlated with the absorption spectrum (Figure 4c). And the AQYs of **PNBN** were 10.9, 3.0, 3.2, and 0.3% at 420, 450, 500, and 550 nm, respectively (Figure 4d). The highest AQY at 420 nm is attributed to its greater penetration capability at shorter wavelength of 420 nm light.^[^ ^54^ ^]^ The tested AQYs are in good agreement with the absorption spectrum of **PNBN**, indicating that most of the photons captured by the material are involved in photoreaction. These results suggest that the introduction of N and B in the conjugated backbone may contribute to the separation and transfer of photo‐generated carriers, enhancing the photocatalytic hydrogen production activity of the polymer. Meanwhile, the photostability of polymer **PNBN** was further verified by cycling experiments. Although the structure of the sample **PNBN** showed some changes after 21 h of photocatalysis, its HER was still much higher than those of **PNCC** and **PNNC** (Figure S33, Supporting Information).

**Figure 5:**
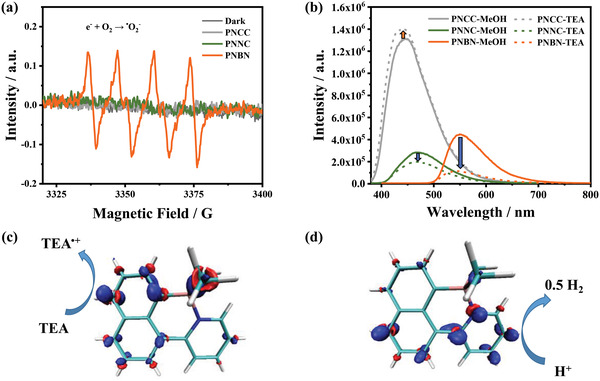
a) EPR spectra of polymers **PNCC**, **PNNC**, and **PNBN** for detection of ^•^O_2_^−^ radicals in a methanol dispersion. b) Fluorescence quenching spectra of polymers **PNCC**, **PNNC**, and **PNBN**. Insets: schematic representation of the transfer of excitation‐generated holes to a sacrificial donor (TEA). Isosurfaces of the charge density difference between the: c) hole/d) electron polaron and ground state of **MNBN1‐DFT**.

To gain a better understanding of the photocatalytic process, the transient absorption spectroscopy (TAS) of the polymers were tested. Figures S38–S40 (Supporting Information) show the transient spectra of suspensions of **PNCC**, **PNNC**, and **PNBN** (H_2_O/MeOH/TEA) from 1 ps to 5 ns after excitation at 400 nm. After excitation, the negative transient signal of the **PNBN** between 450 and 600 nm overlaps spectrally with the polymer photoluminescence shown in Figure 1b, and it is therefore mainly classified as stimulated emission from polymer excitons. For all three polymers, they exhibit prominent new positive absorption features around 691 nm being assigned to the active species of hydrogen evolution on the polymer.^[^ ^51^ ^]^ In addition, the results of the overall decay kinetics of the polymers at 691 nm show that the lifetime of **PNBN** at 691 nm is considerably longer (223.4 ps) compared with **PNCC** (81.7 ps) and **PNNC** (83.2 ps), suggesting that **PNBN** with B←N bonds is more conducive to photocatalysis. Electron paramagnetic resonance (EPR) experiments were carried out under light in order to further confirm the redox properties of the polymers. According to the results shown in **Figure** **5**a, no signal of DMPO‐^•^O_2_^−^ in **PNCC** and **PNNC** is detected after light irradiation. The typical signal of ^•^O_2_^−^ was found in **PNBN**, indicating the photo‐generated charge carriers in **PNBN** exhibited strong reduction ability during the photocatalytic process. Unfortunately, we did not detect signals for hydroxyl radicals in any of the three polymers (Figure S41, Supporting Information). This may be due to the difficulty of the polymers to reach the potential for the generation of H_2_O/^•^OH (1.99 eV vs RHE) or OH^−^/^•^OH (2.38 eV vs RHE) radicals.^[^ ^55^, ^56^ ^]^

In order to clearly verify the high hydrogen production activity of **PNBN** in the H_2_O/CH_3_OH/TEA system, we performed fluorescence quenching experiments in solution. As shown in Figure 5b, **PNBN** exhibited a strong fluorescence peak in the H_2_O/CH_3_OH mixture at *λ* = 550 nm. When TEA was added to the **PNBN** suspension, the fluorescence intensity of **PNBN** decreased dramatically. This suggests that the presence of TEA can inhibit excitons recombine via the radiative pathway. At the same concentration, **PNCC** and **PNNC** still maintain high fluorescence intensities. This is clearly not conducive to charges transfer under the photocatalytic reaction system. The fluorescence quenching results and photocatalytic experiments confirm that the presence of sacrificial agent can greatly influence the electron‐hole separation/recombination pathway of **PNBN**.

In addition, to confirm the role of B←N bond in the photocatalytic process, we calculated the Gibbs free energy change of HER on the B and N sites. As can be observed in Figure S53 (Supporting Information), there is a distinct difference in ΔG_H_ when the N or B sites of **PNNC** and **PNBN** are hydrogenated. The hydrogen‐binding free energy at the N and B site of **PNBN** is 0.62 and 1.67 eV higher than that of the N site of **PNNC**, respectively, indicating a high overpotential of HER. These results suggest that the formation of B←N bonds in the polymer structure does not lead to stronger hydrogen adsorption like platinum. The B←N coordination bond mainly contributes to improve the planarity of the backbone and enhance the built‐in electric field, thus promoting the separation and migration efficiency of the photo‐generated carriers.

To investigate the potential reaction sites of **PNBN**, the electron density difference between the hole/electron polaron and ground state of the polymers fragment were calculated. The charge density differences of both **MNCC1‐DFT** and **MNNC1‐DFT** show a relatively uniform distribution (Figures S54–S55, Supporting Information). In contrast, the electron density difference between the electron/hole polaron and ground state of **MNBN1‐DFT** is concentrated on the pyridine ring and the naphthyl group, which were obviously favorable for the redox reaction (Figures 5c,d). Furthermore, the electron distribution of the electron density difference between the first excited state and the ground state to evaluate the trend of electron leap. According to the simulation results (Figure S59, Supporting Information), electrons of the **PNBN** are excited mainly from the naphthalene group (yellow region) and finally leap to the pyridine ring (purple region). When these light‐generated electrons and holes have a sufficiently high driving force, they undergo proton reduction or oxidation at the expense of the electron donor, respectively. In other words, the reduction reaction of water is more likely to occur on the pyridine ring of **PNBN**, while the oxidation reaction with the sacrificial agent is more likely to occur on the naphthyl group. These results suggest that a rapid redox reaction can be achieved on the backbone of **PNBN**, leading to efficient hydrogen evolution.

## 3 Conclusion

In summary, a D–A‐type conjugated polymer photocatalyst with B←N coordination bonds was successfully synthesized. The B←N coordination bonds significantly improved the planarity and rigidity of the conjugated framework and established a strong built‐in electric field through the change of electronic structure. This resulted in the polymer **PNBN** exhibiting excellent hydrogen evolution performance with HER of 217.4 µmol h^−1^ and AQY_420_ = 10.9%, which is much higher than most of the reported organic conjugated polymer photocatalysts. These results imply that the construction of polar bonds provides a new approach for the design and development of photocatalysts.

## Conflict of Interest

The authors declare no conflict of interest.

## Supporting information

Supporting Information

## Data Availability

The data that support the findings of this study are available in the supplementary material of this article.
