# Supplementary material for: Enhanced Built‐in Electric Field Promotes Photocatalytic Hydrogen Performance of Polymers Derived from the Introduction of B←N Coordination Bond
Source: Adv Sci (Weinh). 2022 Oct 26;9(35):2204055. doi: 10.1002/advs.202204055 (PMC9762295; doi:10.1002/advs.202204055)
Supplement: Supplementary file 1 — Supporting Information [file ADVS-9-2204055-s001.pdf]

ADVANCED SCIENCE
Open Access

Supporting Information

for *Adv. Sci.*, DOI 10.1002/advs.202204055

Enhanced Built-in Electric Field Promotes Photocatalytic Hydrogen Performance of Polymers Derived from the Introduction of B←N Coordination Bond

Chenglong Ru, Peiyan Chen, Xuan Wu, Changjuan Chen, Jin Zhang, Hao Zhao, Jincai Wu and Xiaobo Pan**

Supporting Information

Enhanced Built-in Electric Field Promotes Photocatalytic Hydrogen Performance Derived from the Construction of B←N Coordination Bond

Chenglong Ru, Peiyan Chen, Xuan Wu, Changjuan Chen, Jin Zhang, Hao Zhao, Jincai Wu, and Xiaobo Pan**

C. Ru, P. Chen, X. Wu, J. Zhang, Dr. Zhao, Prof. Dr. J. Wu, Prof. Dr. X. Pan

State Key Laboratory of Applied Organic Chemistry (Lanzhou University), Key Laboratory of Nonferrous Metal Chemistry and Resources Utilization of Gansu Province, College of Chemistry and Chemical Engineering, Lanzhou University, Lanzhou 730000 (P.R. China). E-mail: boxb@lzu.edu.cn

Dr. Zhao

School of Physics and Electronic Information, Yantai University, 30 Qingquan Road, Yantai 264005, China. E-mail: zhaohaolc@126.com

Dr. Chen

College of Chemistry and Pharmaceutical Engineering, Huanghuai University, Zhumadian 463000, People's Republic of China.

Prof. Dr. X. Pan

Northwest Institute of Eco-Environment and Resources, Chinese Academy of Sciences, Lanzhou 730000 (P.R. China); Key Laboratory of Petroleum Resources Research, Gansu Province, Lanzhou 730000 (P.R. China).

General Methods

All reactions and manipulations were carried out under an argon atmosphere by using standard Schlenk techniques or an inter-atmosphere glovebox. Prior to use CH_2Cl_2, Et_2O and toluene were dried by refluxing and degassed by applying three freeze-pump-thaw cycles. $CDCl_3$ was dried by 4 Å molecular sieve (2-3 days). All chemicals (reagents and solvents) were obtained from commercial suppliers (Energy Chemical, Heowns) and directly used without further purification.

Instrumentations

The ^1H and ^{13}C NMR spectra were performed using a JEOL JNM-ECS-400 at room temperature in ppm downfield from TMS. Solid-state ^{13}C CP/MAS NMR spectra were recorded at room temperature on an Agilent-600MHz solid state NMR spectrometer, operating at LARMOR frequencies of 150.95 MHz. The samples were contained in a 4 mm zirconium (IV) oxide rotor, which was mounted in a standard double resonance probe at a MAS frequency of 10 kHz. The ^{13}C chemical shifts were referenced relative to tetramethylsilane. A standard cross-polarization sequence with a 3 ms ramped contact pulse. The 960 (**PNCC**), 2710 (**PNNC**) and 2742 (**PNBN**) scans were accumulated with a 3 s cycle delay, respectively. X-ray photoelectron spectroscopy (XPS) was performed on an Axis Supra. Transmission FT-IR spectra were recorded on a Bruker Vertex 70 at room temperature with KBr pellets. Powder X-ray diffraction (PXRD) patterns were measured using X-ray diffraction (XRD, X'pert pro, Philips) which equipped with Cu Kα radiation (λ = 1.54056 Å) at the scattering angle 2θ between 3–50°. The surface morphologies of the polymers were acquired by scanning electron microscopy (SEM) on a Carl Zeiss, Ultra Plus, Germany, operating at 5 kV. The high resolution TEM (HRTEM) images of the samples were characterized by transmission electron microscope (TECNAI G2 TF20, USA). Thermogravimetric analysis (TGA) was performed under nitrogen atmosphere on a NETZSCH STA 449F3 instrument with temperature increase from 25 °C to 700 °C at a heating rate of 10 °C min^{-1}. UV-Vis absorption spectra were recorded at room temperature on Agilent Carry 5000 UV-Vis-NIR spectrometer. Photoluminescence (PL) spectroscopy were collected with a photoluminescence spectrophotometer Edinbergh FLS920 at room temperature. Time-correlated single photon counting (TCSPC) experiments were performed on an Edinbergh FLS920 fluorescence spectrophotometer with picosecond pulsed LED excitation sources and a R928 detector. Decay times were fitted in the F900 software using suggested lifetime estimates. The weighted average lifetime calculated as $\tau_{AVG} = \sum_{i=1}^{n} \alpha_i \tau_i$. Fluorescence quenching experiments were performed in 0.002 M

(H_2O/MeOH=1/1 or H_2O/MeOH/TEA=1/1/1) suspension with a photoluminescence spectrophotometer Edinbergh FLS920. All data were collected at room temperature for samples without deoxygenation. The slit widths were 1 nm/1 nm and the scanning speed was 1200 nm/min. Atomic force microscope (AFM) images of the samples were performed on a Dimension Lcon. Zeta potential was performed on a 90Plus Pals. Dynamic light scattering (DLS) measurements were performed on a Malvern Mastersizer 2000 Particle Sizer. Transient photocurrent responses and electrochemical impedance spectra were recorded using a CHI-760E electrochemical workstation in a standard three electrode configuration. The polymers (4.0 mg) was dispersed in ethanol (0.5 mL) containing 1 % nafion by ultrasound for 1 hour. The work electrodes were prepared via drop-casting the mixture 20 μL onto the surface of FTO glass substrate electrode and then being filmed at 80 ºC in vacuum oven. The Pt plate served as the counter electrode, and a saturated Ag/AgCl electrode as a reference electrode. A 0.5 M Na_2SO_4 solution was used as the electrolyte. A 300 W Xenon lamp equipped with a 420 nm cut-off filter (100 mW cm^{-1}) was used as the light source in the photocurrent response measurement. Gas Chromatography - Thermal Transformation - Isotope Ratio Mass Spectrometer (GC-TC-IRMS) was performed under helium atmosphere on a ThermoFisher Trace1310-Delta V Advantage with a capillary column (25 m × 0.32 mm × 1.0 μm; PoraPLOT Q). GC was used under the following conditions: Split injection, split ratio 10:1; Initial temperature 50°C, constant for 3 min, then ramped up to 190°C at 30°C min^{-1}. The femtosecond - transient absorption (fs-TA) measurements were performed on a Legent Elite. A Ti:sapphire femtosecond laser system provided laser pulses. A regenerative amplifier (Coherent, Legend Elite) seeded with a mode-locked Ti:sapphire laser (Coherent, Chameleon) delivered laser pulses at 800 nm (120 fs, 1 kHz). The fs-TA was recorded by a femtosecond transient absorption spectrometer (ultrafast, HELIOS) based on pump–probe technology. The 800 nm laser was attenuated and focused into a 3 mm sapphire plate to generate the probe pulses. The 400 nm pump laser was obtained using thin BBO crystal plates for second harmonic generation. The temporal resolution between the pump and the probe pulses was determined to be ~150 fs (FWHM). The liquid samples () were measured in a 2 mm quartz cell. The excitation pulsed energy was ~50 nJ per pulse, as measured at the sample site. Electron paramagnetic resonance (EPR) measurements were carried out on a CIQTEK EPR200M spectrometer. The EPR spectrum was obtained at room temperature with the microwave power 2.42 mW and frequency 9.429 GHz. The radical of polymers (1.0 mg mL^{-1}) were detected with 5,5-dimethyl-1pyrroline N-oxide (DMPO, 100 mM) as the capture agent (irradiation time

of 1 min). Palladium content was determined via inductively coupled plasma mass spectrometry (ICP-MS), which carried out in an Elementar Vario EL III Carlo Erba 1108 elemental analyzer by ashing and digesting the sample in a mixture of concentrated HNO_3 : HCl = 1:3.

X-ray Crystallography

The data were collected with a SuperNova (Dual) X-ray diffractometer that equipped with Cu/Mo Kα radiation (λ = 1.54184/0.71073 Å) at different temperature. Data reduction was performed using CrysAlisPro (Version 1. 171. 37. 35). The data sets were corrected by empirical absorption correction using spherical harmonics, implemented in SCALE3 ABSPACK scaling algorithm. Crystal structure was solved by direct methods using Olex 2-1.2. Subsequent difference Fourier analyses and least squares refinement with SHELXL-2014/7 program package allowed for the location of the atom positions. Non-hydrogen atoms were refined with anisotropic displacement parameters during the final cycles. All hydrogen atoms were found in difference maps and refined using a riding model. More details on the crystallographic studies as well as atomic displacement parameters are given in CIF files. The crystallographic details for compound **NBNME-Br-BO** is summarized in Table S1. The data have been deposited in the Cambridge Crystallographic Data Centre (CCDC), deposition numbers CCDC 2183658 for compound **NBNME-Br-BO**.

Photocatalytic activity measurements

A flask was charged with the polymer powder (10 mg), a 1:1:1 vol. mixture of water, triethylamine, and methanol (50 mL). The resulting suspension was ultrasonicated until the photocatalyst was dispersed before degassing by N_2 bubbling for 30 minutes. The accumulated amount of evolved gases was monitored every 60 min using a gas chromatograph (Varian 450-GC) equipped with a thermal conductive detector (TCD). The light source was a 300 W Xe lamp with a cut-off filter applied to generate visible light (λ > 420 nm). Hydrogen was detected with a TCD detector, referencing against standard gases with known concentrations of hydrogen. Hydrogen dissolved in the reaction mixture was not measured, and the pressure increase generated by the evolved hydrogen was neglected in the calculations.

Apparent quantum yield measurements

The apparent quantum yield (AQY) for H_2 evolution was measured using monochromatic visible 420 nm, 450 nm, 500nm and 550 nm. The irradiation energy measured by calibration power meter was 5.5, 14.8, 17.0 and 17.5 mW cm^{-1}, respectively. The irradiation area was controlled as 13.2 cm^2.

Depending on the amount of hydrogen produced by the photocatalytic reaction in an average of 3 h, the AQY was calculated as the follow equation:

$$AQY = \frac{N_e}{N_p} \times 100\%$$
$$= \frac{2 \times M \times N_A}{E_{total}/E_{photon}} \times 100\%$$
$$= \frac{2 \times M \times N_A}{(S \times P \times t)/\left(h \times \frac{c}{\lambda}\right)} \times 100\%$$
$$= \frac{2 \times M \times N_A \times h \times c}{S \times P \times t \times \lambda} \times 100\%$$

Where, M is the amount of H_2 (mol), N_A is Avogadro constant (6.022×10^{23} /mol), h is the Planck constant (6.626×10^{-34} J·s), c is the speed of light (3×10^8 m/s), S is the irradiation area (cm^2), P is the intensity of irradiation light (W/cm^2), t is the photoreaction time (s), λ is the wavelength of the monochromatic light (m).

Experimental Section

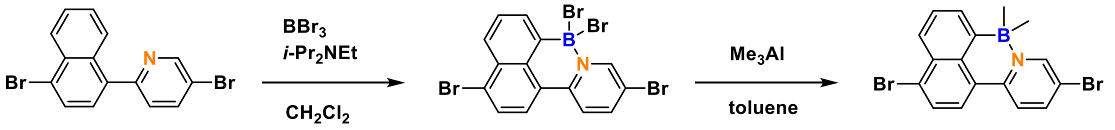

Synthesis of 5-bromo-2-(4-bromo-2-(dibromoboranyl)naphthyl)pyridine (NBNBr$_2$-Br$_2$).

To a stirred solution of 5-bromo-2-(4-bromonaphthyl)pyridine (3.48 g, 9.58 mmol) and *i*-Pr_2NEt (1.6 mL, 9.20 mmol) in CH_2Cl_2 (50 mL) at -78 °C, BBr_3 (1.0 M in CH_2Cl_2, 20 mL, 20.00 mmol) was added dropwise. After being stirred at room temperature for 12 h, saturated K_2CO_3 aqueous solution was added to the reaction mixture. The formed precipitate was collected via filtration and washed with water and acetone to afford a **NBNBr$_2$-Br$_2$** as a pale yellow solid (4.74 g, 8.90 mmol, 93%). (^{1}H and ^{13}C{^{1}H} NMR could not be measured due to the low solubility.)

Synthesis of 5-bromo-2-(4-bromo-2-(dimethylboranyl) naphthyl)pyridine (NBNME-Br$_2$).

To a stirred solution of **NBNBr$_2$-Br$_2$** (2.66 g, 5.00 mmol) in toluene (50 mL) at room temperature was added $AlMe_3$ (1.1 M in hexane, 10 mL, 11.00 mmol). After being stirred at this temperature for 2 h, the reaction was quenched by adding water. The organic layer was separated and extracted with ethyl acetate (twice), washed with water (once), brine (once), and dried over $MgSO_4$ and concentrated. The residue was purified by preparative thin layer chromatography on silica gel to afford **NBNME-Br$_2$**

(1.85 g, 4.60 mmol, 92 % yield). ^{1}H NMR (400 MHz, $CDCl_3$, ppm): δ 8.90 (s, 1H), 8.17 (d, J = 8.0 Hz, 1H), 8.04 (d, J = 8.0 Hz, 2H), 7.93 (d, J = 8.0 Hz, 1H), 7.80 (d, J = 8.0 Hz, 1H), 7.71 (d, J = 8.0 Hz, 1H), 7.65 (t, J = 8.0 Hz, 1H), 0.25 (s, 6H). $^{13}C\{^{1}H\}$ NMR (100 MHz, $CDCl_3$, ppm): δ 151.20, 146.77, 141.90, 131.67, 131.41, 130.60, 129.06, 128.70, 128.55, 126.49, 123.74, 123.34, 123.28, 118.75, 15.89.

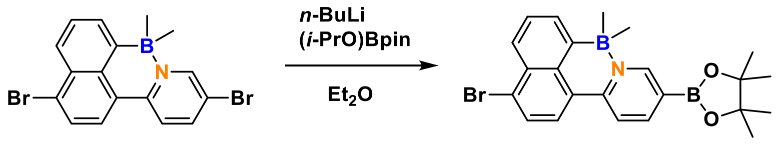

NBNME-Br₂ **NBNME-Br-BO**

Synthesis of 2-(4-bromo-2-(dimethylboranyl)naphthyl)-5-(4,4,5,5-tetramethyl-1,3,2-dioxaborolan-2-yl)pyridine (NBNME-Br-BO).

To a stirred solution of **NBNME-Br$_2$** (1.21 g, 3.00 mmol) in Et_2O (30 mL) at -78 ˚C was added n-BuLi (1.6 M in hexane, 1.94 mL, 3.10 mmol). After being stirred for 1 h at this temperature, i-PrOBpin (1.12 g, 6.00 mmol) was added and the reaction mixture was stirred for 12 h at room temperature. After quenching with water, the organic layer was separated and the aqueous layer was then extracted with ethyl acetate (twice). The organic layer was combined and washed with water (once), brine (once), dried over $MgSO_4$ and concentrated. The residue was purified by preparative thin layer chromatography on silica gel to afford **NBNME-Br-BO** (1.13 g, 2.52 mmol, 84% yield). ^{1}H NMR (400 MHz, $CDCl_3$, ppm): δ 9.12 (s, 1H), 8.30 (d, J = 8.0 Hz, 1H), 8.25 (d, J = 8.0 Hz, 1H), 8.02 (t, J = 8.0 Hz, 2H), 7.81 (d, J = 8.0 Hz, 1H), 7.74 (d, J = 4.0 Hz, 1H), 7.65 (t, J = 4.0 Hz, 1H),1.39 (s, 12H), 0.27 (s, 6H). $^{13}C\{^{1}H\}$ NMR (100 MHz, $CDCl_3$, ppm): δ 153.63, 151.52, 144.58, 131.73, 131.60, 130.47, 128.85, 127.34, 124.06, 123.07, 1121.38, 85.04, 25.00, 14.31.

Typical procedure of Suzuki–Miyaura coupling polymerization as follows:

A flask was charged with the monomers, DMF and an aqueous solution of K_2CO_3 (2.0 M). The mixture was degassed by bubbling with N_2 for 20 minutes, before $[Pd(PPh_3)_4]$ was added, and heated to 100 °C for 2 days. The mixture was cooled to room temperature and poured into water. The precipitate was collected by filtration and washed with H_2O, methanol, acetone and dichloromethane. Further purification was performed by Soxhlet extraction with methanol and THF.

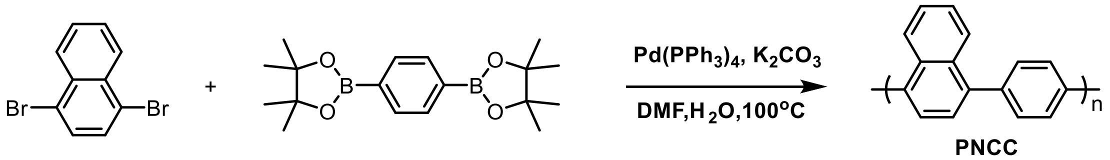

Synthesis of PNCC.

1,4-Dibromonaphthalene (286 mg, 1.0 mmol), 1,4-Benzenediboronic acid bis(pinacol) ester (330 mg, 1.0 mmol), [Pd(PPh_3)$_4$] (40 mg), DMF (30 mL) and an aqueous solution of K_2CO_3 (2 M, 6 mL) were used. After work-up, the product was obtained as off-white solid (190 mg, 82 % yield).

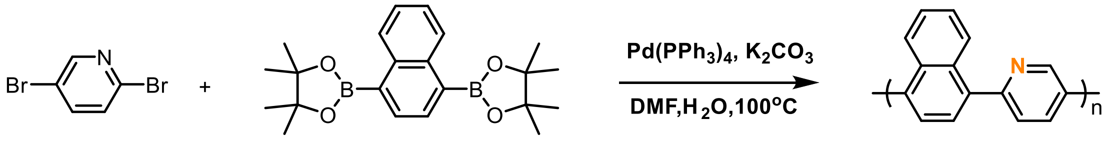

Synthesis of PNNC.

2,5-Dibromopyridine (237 mg, 1.0 mmol), 1,4-Naphthalenediboronic acid bis(pinacol) ester (380 mg, 1.0 mmol), [Pd(PPh_3)$_4$] (40 mg), DMF (30 mL) and an aqueous solution of K_2CO_3 (2.0 M, 6 mL) were used. After work-up, the product was obtained as green solid (179 mg, 77 % yield).

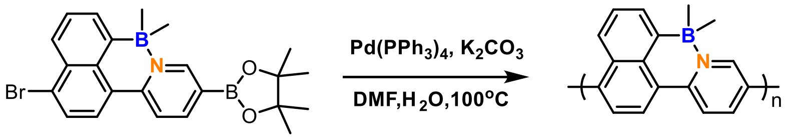

Synthesis of PNBN.

NBNME-Br-BO (450 mg, 1.00 mmol), [Pd(PPh_3)$_4$] (20 mg), DMF (15 mL) and an aqueous solution of K_2CO_3 (2.0 M, 3 mL) were used. After work-up, the product was obtained as laurel-green solid (227 mg, 83 % yield).

Table S1. Single crystal X-ray refinement details for **NBNME-Br-BO** structure refinement.

**NBNME-Br-BO**			
Formula	$C_{23}H_{26}B_2BrNO_2$	α (°)	90°
Mr [g mol^{-1}]	449.98	β (°)	114.402(3)°
Crystal system	*monoclinic*	γ (°)	90°
Space group	$P2_1/n$	V [Å^3]	*2163.96(9)*
Z	4	F(000)	928
μ (mm^{-1})	2.726	Reflections collected	15137
a (Å)	16.7129(4)	GOF	1.075
b (Å)	7.30500(10)	Final R indexes [I≥2σ (I)]	R_1 = 0.0477, wR_2 = 0.1422
c (Å)	19.4633(4)	CCDC Number	2183658

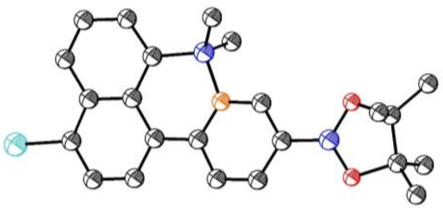

Figure S1. Thermal ellipsoid (50%) and crystal packing drawings of model compounds **NBNME-Br-BO**.

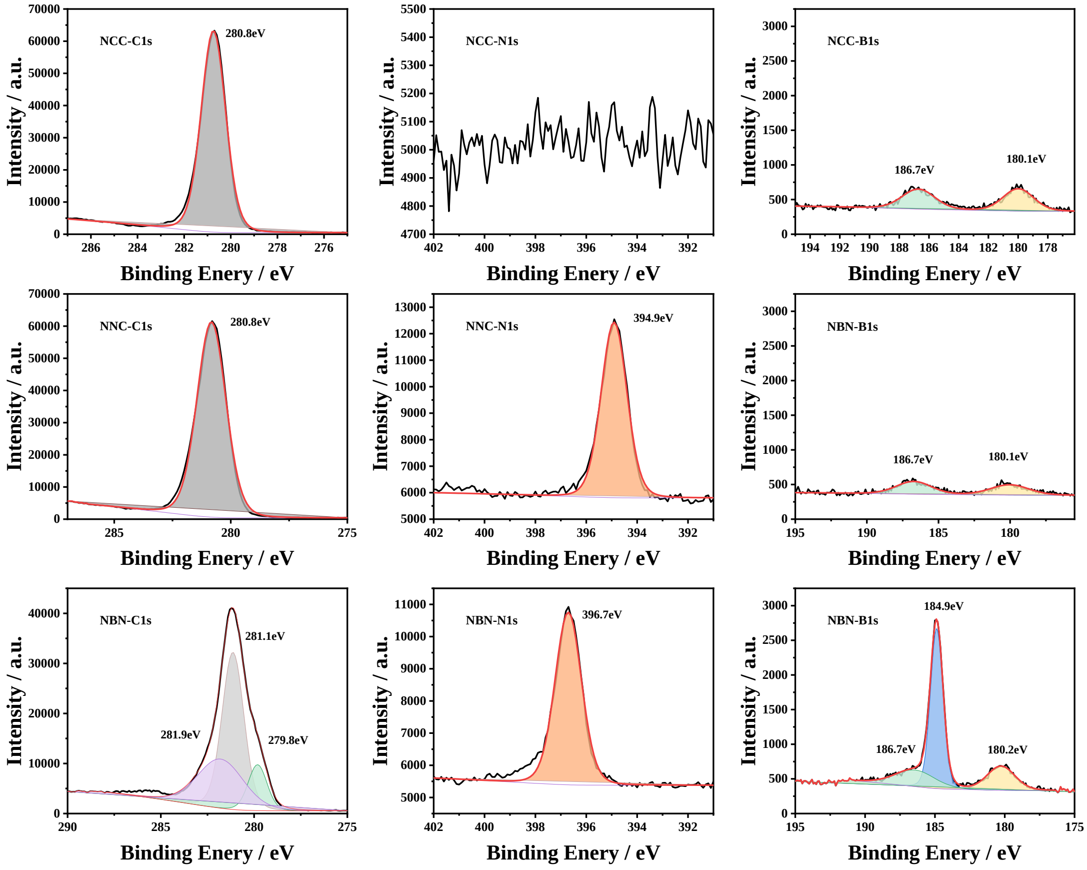

Figure S2. XPS spectra of polymers **PNCC**, **PNNC**, and **PNBN**.

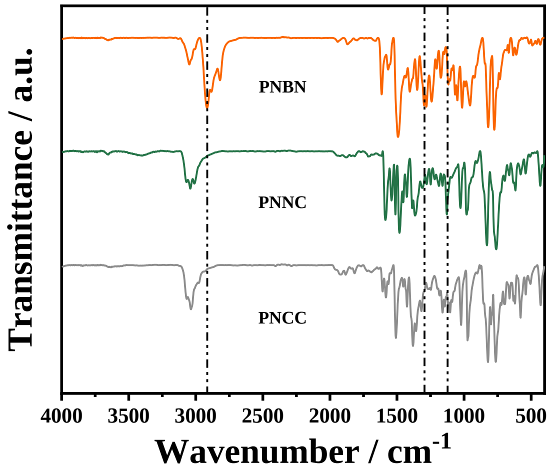

Figure S3. Transmission FT-IR spectra of the polymers **PNCC**, **PNNC**, and **PNBN**.

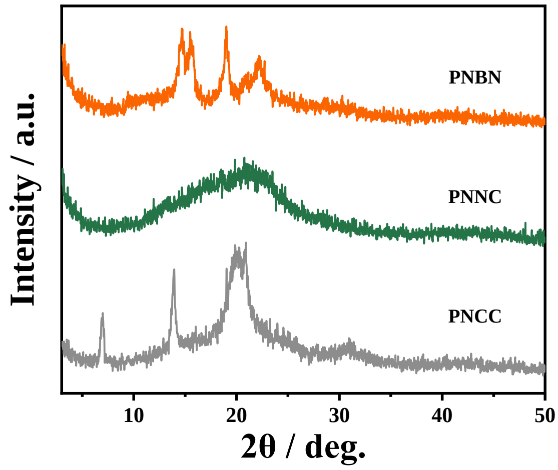

Figure S4. PXRD of polymers **PNCC**, **PNNC**, and **PNBN**.

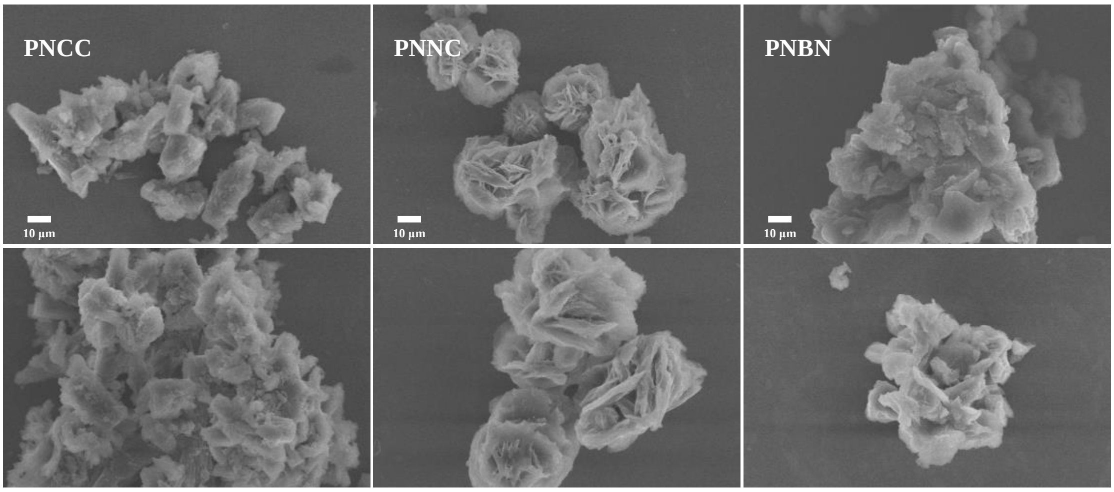

Figure S5. SEM images of polymers before (up) and after photocatalysis (down).

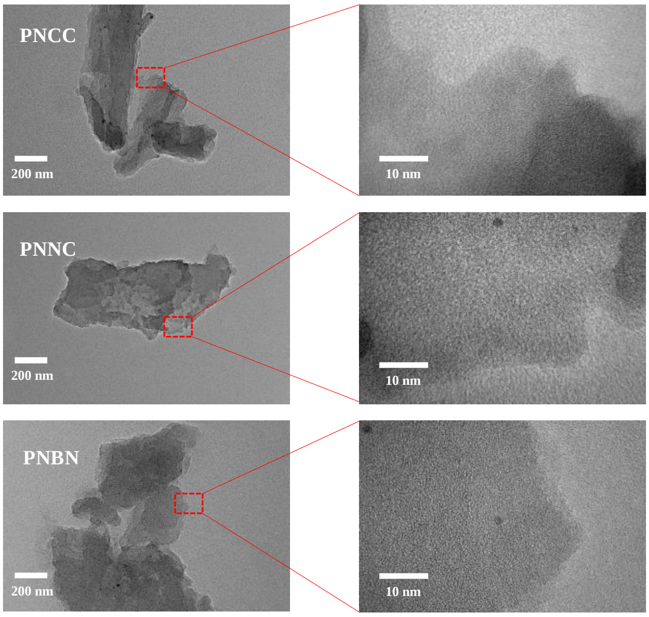

Figure S6. TEM images of polymers **PNCC**, **PNNC**, and **PNBN**.

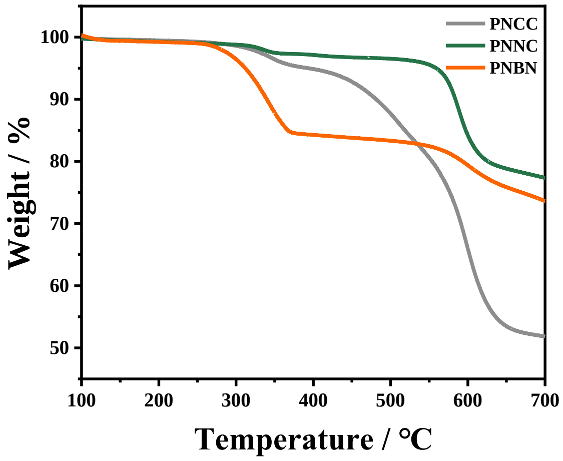

Figure S7. TGA traces of polymers **PNCC**, **PNNC**, and **PNBN**.

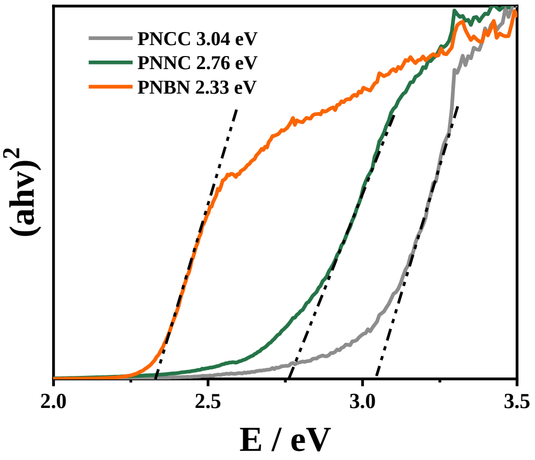

Figure S8. Tauc plots of polymers **PNCC**, **PNNC**, and **PNBN**.

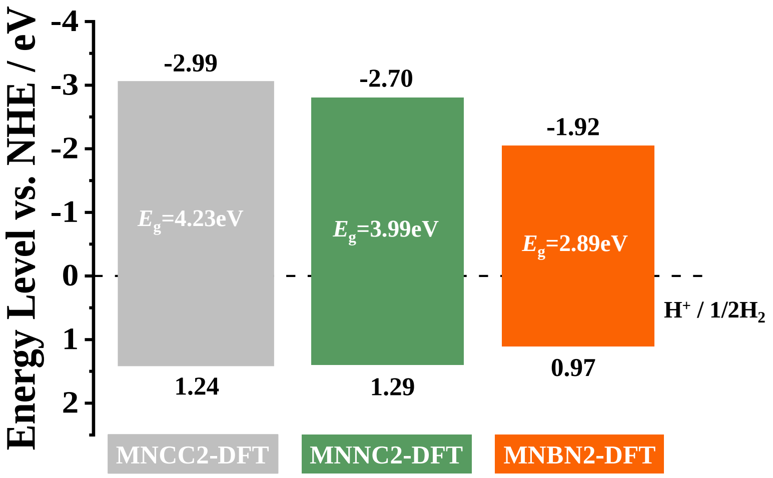

Figure S9. Experimentally determined HOMO and LUMO energy levels of model compounds **MNCC2-DFT**, **MNNC2-DFT**, and **MNBN2-DFT**.

Table S2. DFT calculated geometric structures and molecular orbital diagrams of the fragmental structure of **MNCC1-DFT**, **MNNC1-DFT**, **MNBN1-DFT**, **MNCC2-DFT**, **MNNC2-DFT**, and **MNBN2-DFT**.

	HOMO	LUMO	Fragments
**MNCC1-DFT**	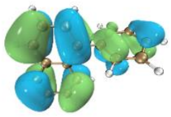	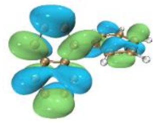	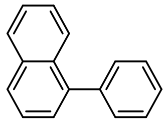
**MNNC1-DFT**	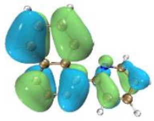	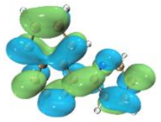	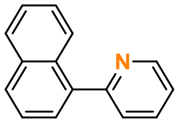
**MNBN1-DFT**	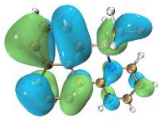	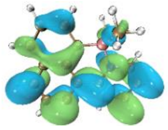	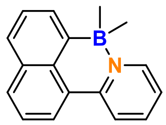
**MNCC2-DFT**	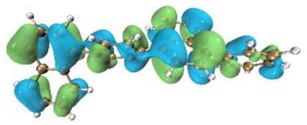	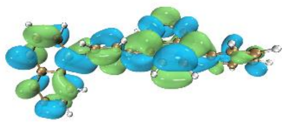	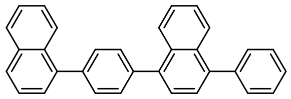
**MNNC2-DFT**	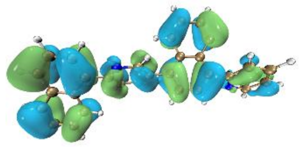	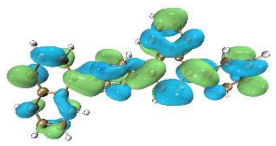	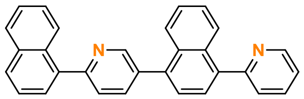
**MNBN2-DFT**	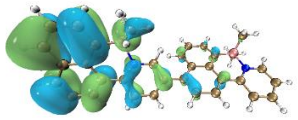	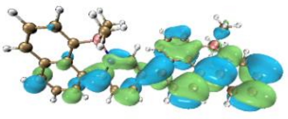	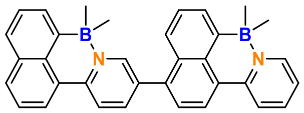

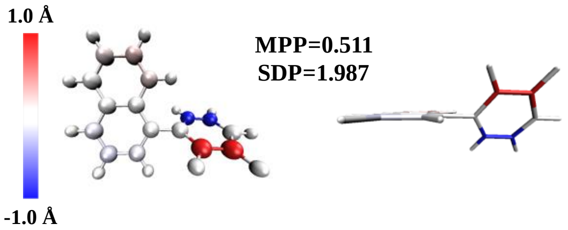

Figure S10. MPP and SDP values for all carbon in the **MNCC1-DFT**. The bluer (redder) the color, the larger the distance of the atom below (above) the fitting plane.

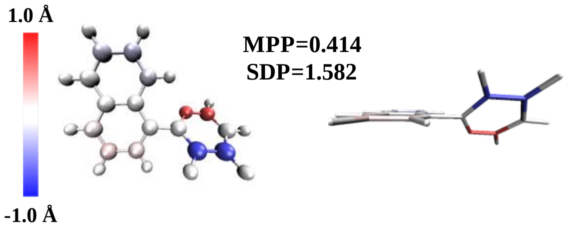

Figure S11. MPP and SDP values for all carbon in the **MNNC1-DFT**.

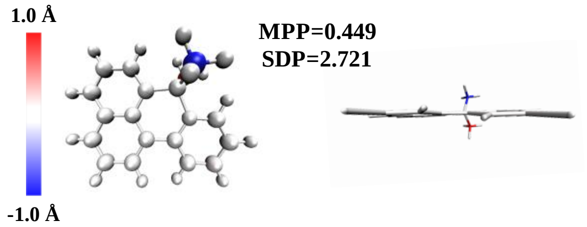

Figure S12. MPP and SDP values for all carbon in the **MNBN1-DFT**.

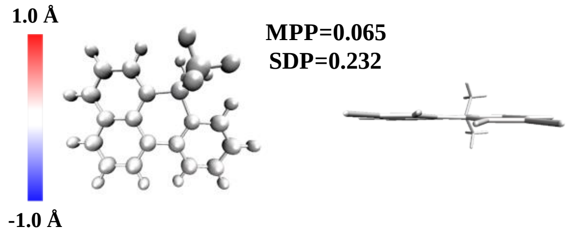

Figure S13. MPP and SDP values of aryl carbon in the **MNBN1-DFT**. (Excluding methyl carbon)

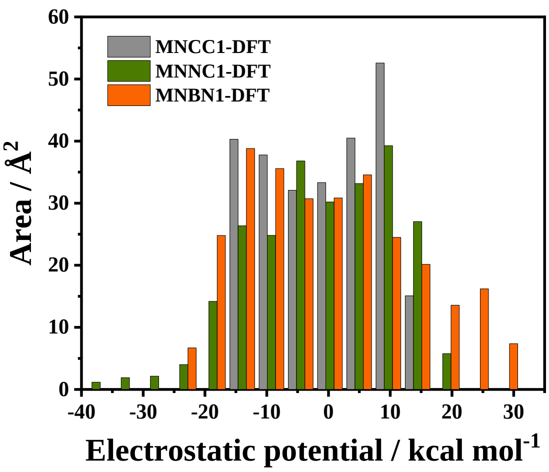

Figure S14. Surface area in each ESP range on the vdW surface of **MNCC1-DFT**, **MNNC1-DFT**, and **MNBN1-DFT**.

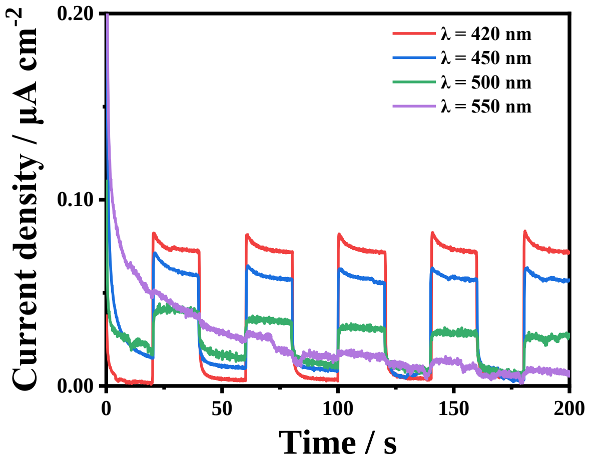

Figure S15. The transient photocurrent responses of **PNBN** at λ = 420, 450, 500, and 550 nm.

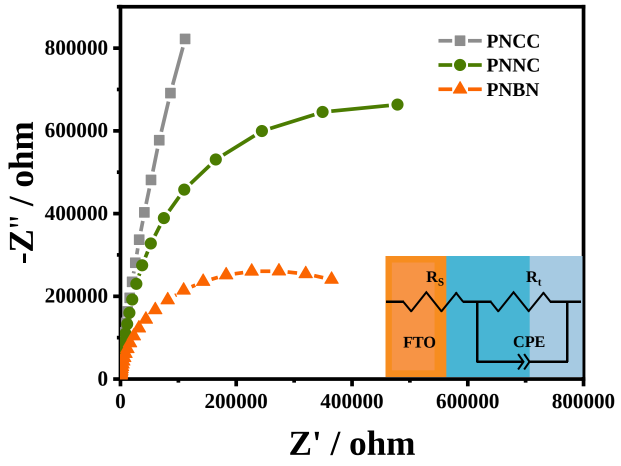

Figure S16. EIS Nyquist plots of polymers **PNCC**, **PNNC**, and **PNBN**.

Table S3. Photophysical properties and HERs of the polymers.

Material	λ_{abs}(nm)	E_g (eV)	Pd(wt%)	HER(μmol h^{-1})[a]
**PNCC**	424	3.04	0.29	0
**PNNC**	468	2.76	0.53	0.66
**PNBN**	547	2.33	0.44	217.4

[a]10 mg of the photocatalyst was suspended in 50 mL of a 1:1:1 mixture solution of water, methanol and triethylamine (TEA) and irradiated by visible light (λ > 420 nm).

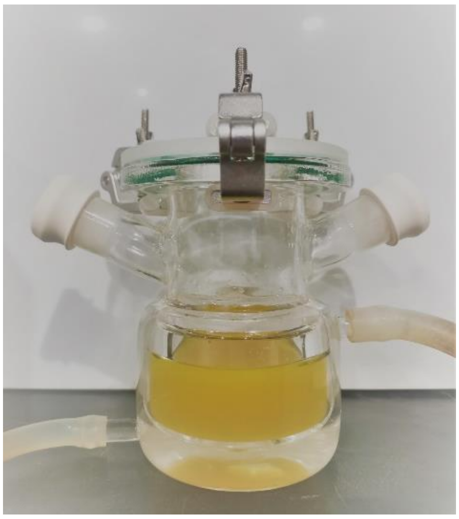

Figure S17. Photograph of the photocatalytic analytical system.

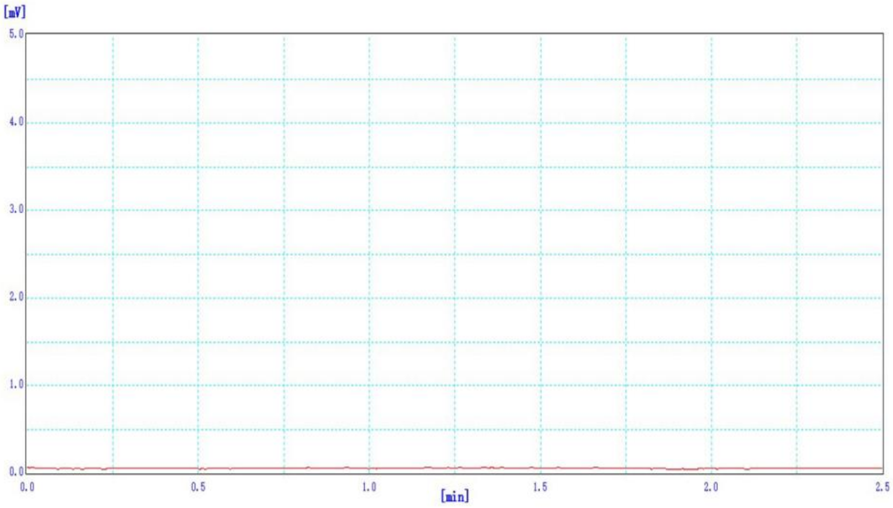

Figure S18. Gas chromatogram of **PNBN** after 0 h illumination.

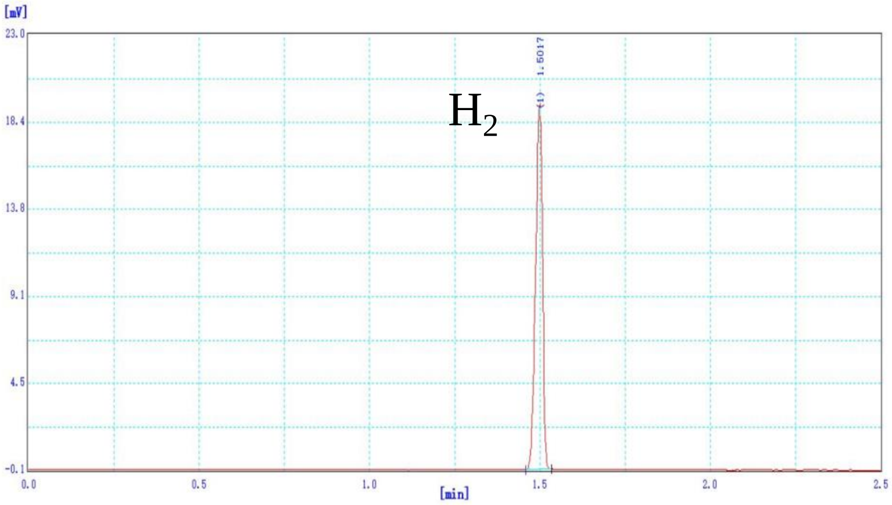

Figure S19. Gas chromatogram of **PNBN** after 5 h illumination.

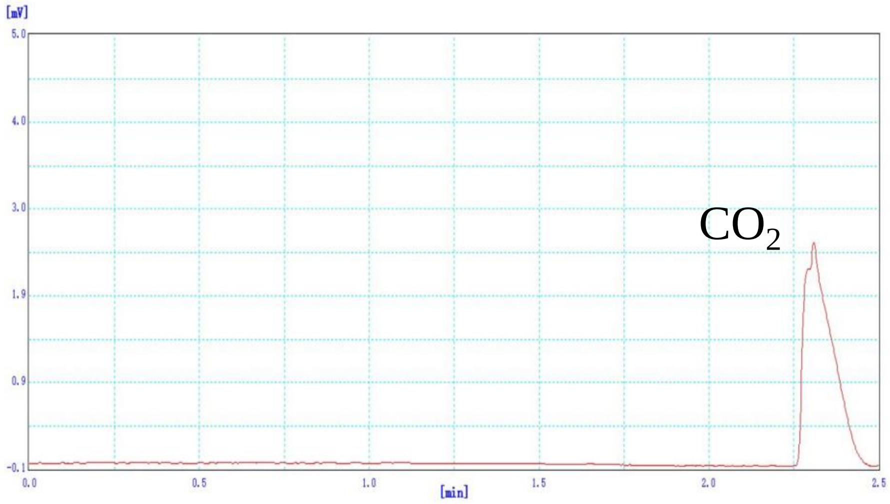

Figure S20. Gas chromatogram of CO_2.

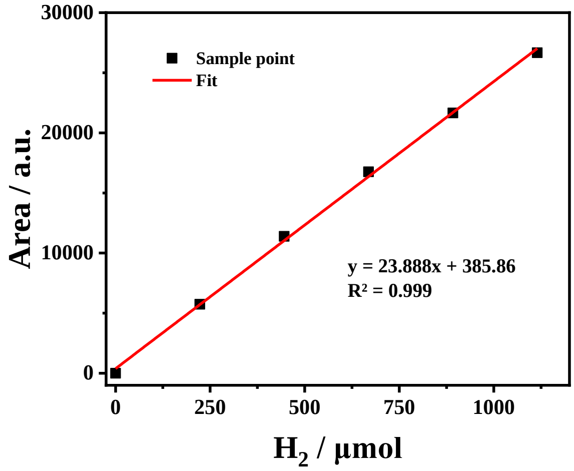

Figure S21. H_2 peak area verse the number of moles of hydrogen and polynomial fit of sample point to be H_2 normalized curve.

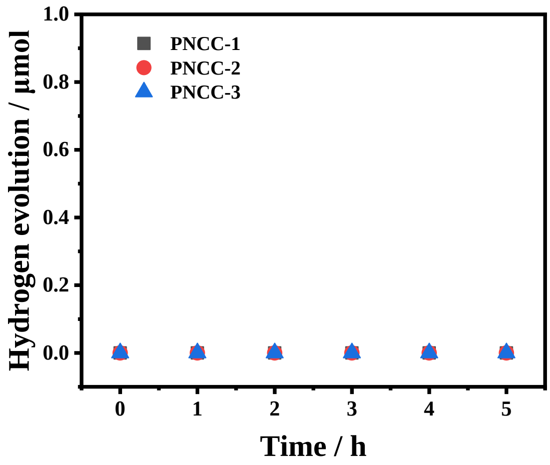

Figure S22. Time-course for photocatalytic hydrogen production using visible light for different batches of **PNCC** (10 mg catalyst in H_2O/CH_3OH/TEA mixture).

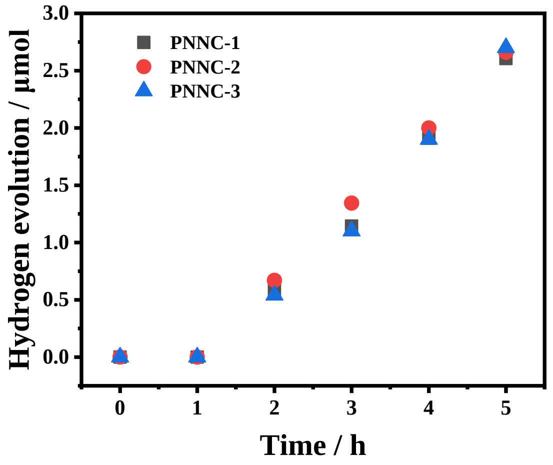

Figure S23. Time-course for photocatalytic hydrogen production using visible light for different batches of **PNNC** (10 mg catalyst in $H_2O/CH_3OH/TEA$ mixture).

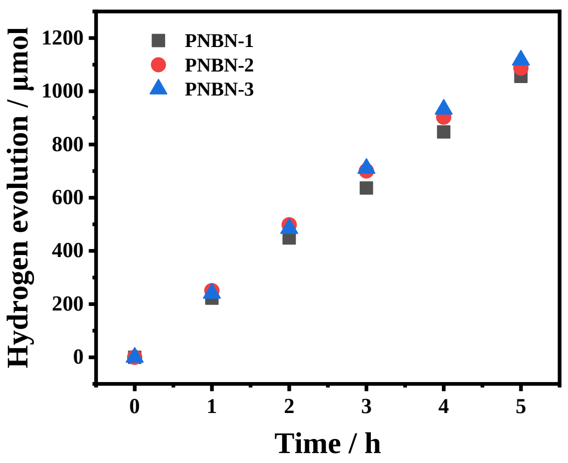

Figure S24. Time-course for photocatalytic hydrogen production using visible light for different batches of **PNBN** (10 mg catalyst in $H_2O/CH_3OH/TEA$ mixture).

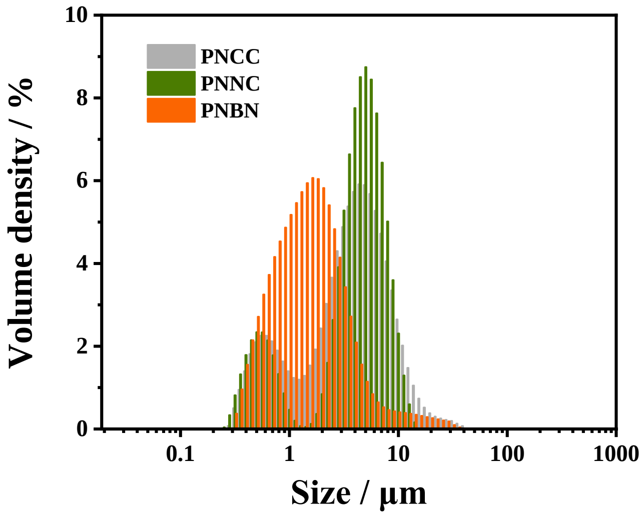

Figure S25. Dynamic light scattering experiments of **PNCC**, **PNNC**, and **PNBN** in methanol.

Table S4. Particle sizes by dynamic light scattering.

Polymer	D[3,2][a]	D[4,3][b]	Dx50[c]
**PNCC**	1.96	5.04	4.05
**PNNC**	2.00	4.76	4.65
**PNBN**	1.25	2.43	1.57

[a] Surface area mean diameter (Sauter mean diameter); [b] Volume mean diameter; [c] 50th percentile of particle size volume distribution.

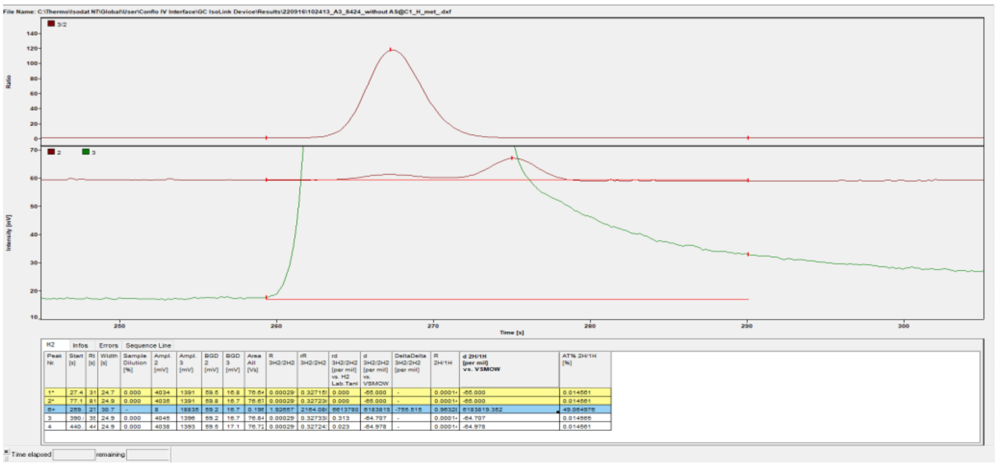

Figure S26. Isotope ratio mass spectra of D and H.

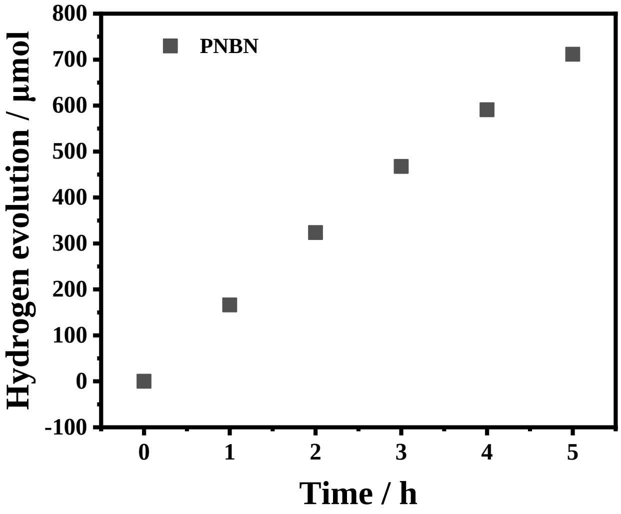

Figure S27. Photocatalytic hydrogen evolution of **PNBN** from H_2O/TEA (8 vol. %) mixtures under λ > 420 nm irradiation.

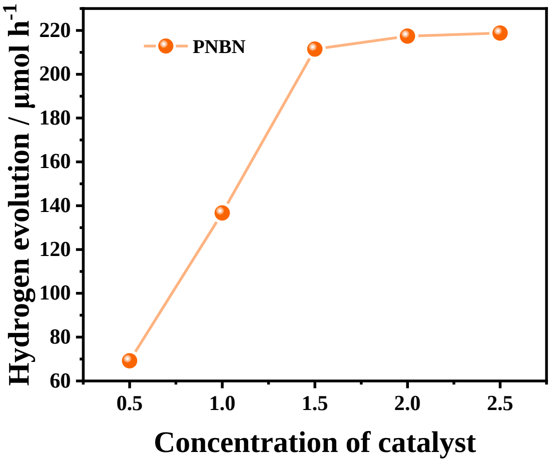

Figure S28. Dependence of the amount of H_2 production on photocatalyst concentration for **PNBN**.

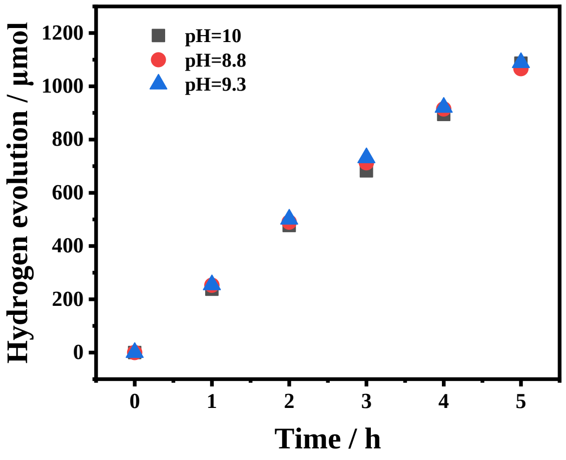

Figure S29. Time-course for photocatalytic hydrogen production using visible light for **PNBN** (10 mg catalyst, PH=8.8, 9.3, and 10).

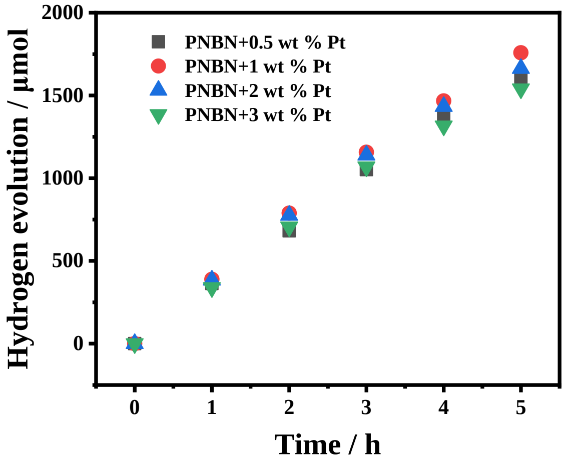

Figure S30. Time-course for photocatalytic hydrogen production using visible light for **PNBN** with 0.5, 1, 2, and 3 wt % Pt cocatalyst (10 mg catalyst in H_2O/CH_3OH/TEA mixture).

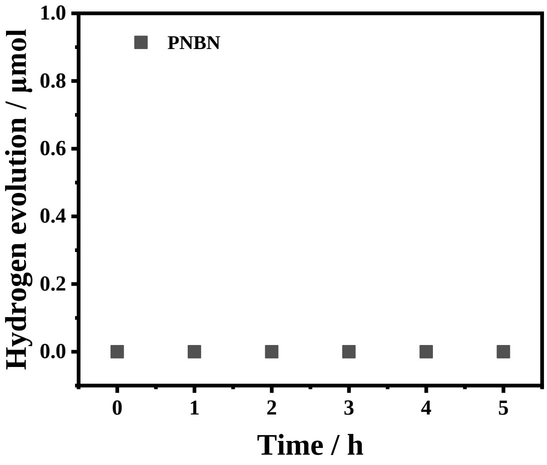

Figure S31. Photocatalytic hydrogen evolution of **PNBN** from H_2O/CH_3OH mixtures under λ > 420 nm irradiation.

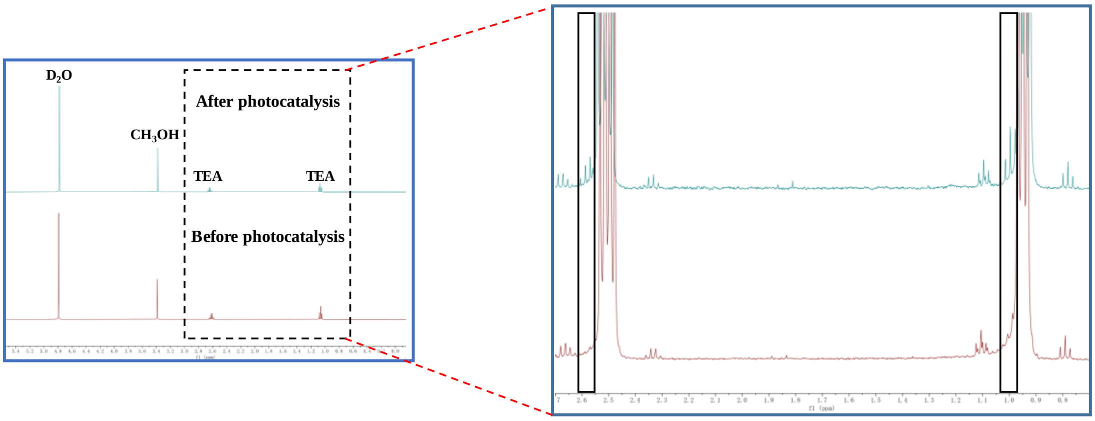

Figure S32. ^1H NMR spectra of system (H_2O+CH_3OH+TEA+catalyst) were collected before and after 5 h illumination.

Table S5. The dependence of the gas evolution rates on the photocatalyst mass, solution pH and Pt co-catalyst loading.

Photocatalyst (mg)	Concentration (g L^{-1})	pH[a]	Pt (wt %)	HER (µmol h^{-1})
0	0	10	0	0
2.5	0.05	10	0	69.2
5	0.1	10	0	136.7
7.5	0.15	10	0	211.5
10	0.2	10	0	217.4
12.5	0.25	10	0	218.8
10	0.2	8.8	0	219.2
10	0.2	9.3	0	215.4
10	0.2	10	0.5	327.0
10	0.2	10	1	354.1
10	0.2	10	2	337.9
10	0.2	10	3	314.1

[a] pH was regulated by triethylamine hydrochloride.

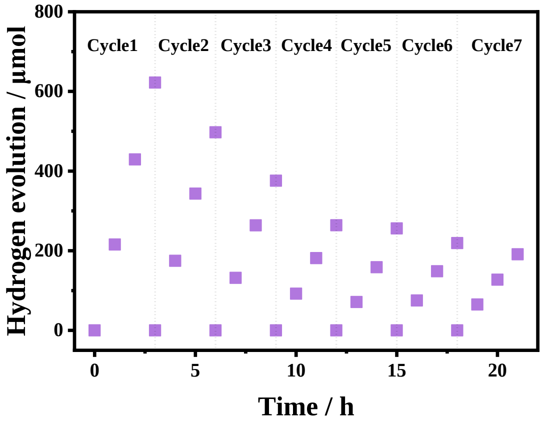

Figure S33. Photostability and reusability using **PNBN** as a photocatalyst under visible-light illumination (λ > 420 nm).

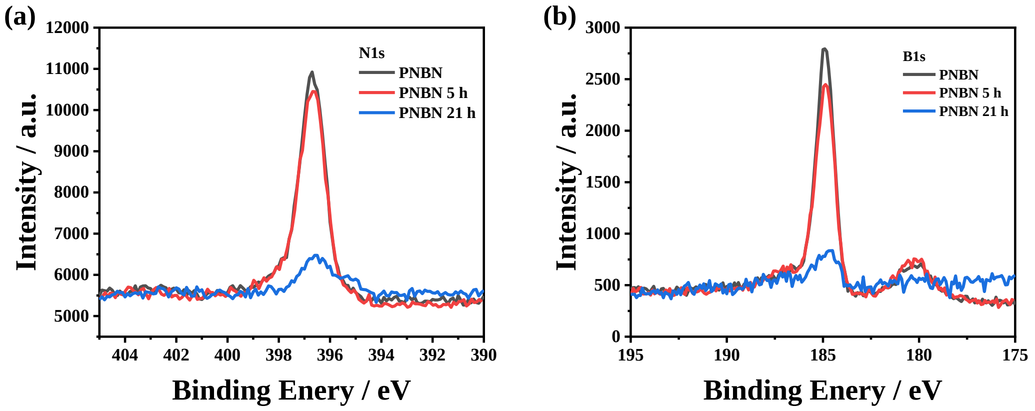

Figure S34. XPS (a) N 1s and (b) B 1s spectra of **PNBN** before and after photocatalysis.

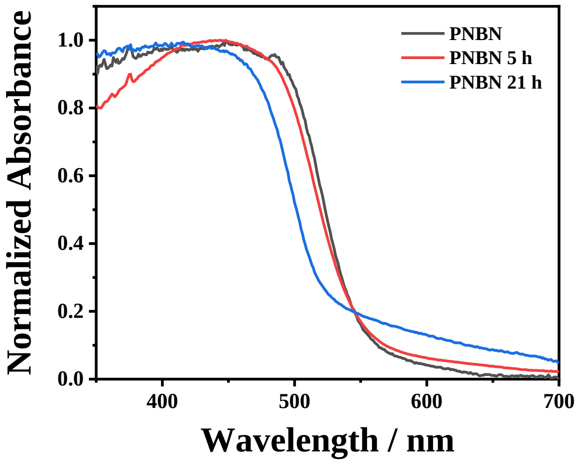

Figure S35. UV−vis absorption of **PNBN** before and after photocatalysis.

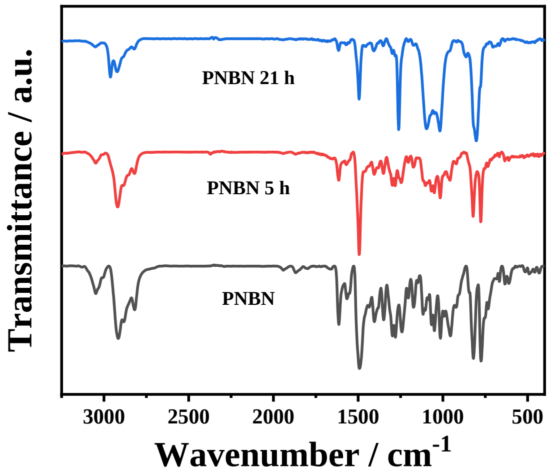

Figure S36. FTIR spectra of **PNBN** before and after photocatalysis.

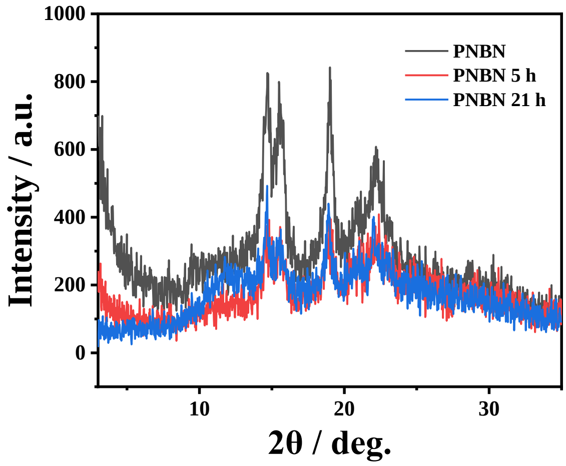

Figure S37. XRD pattern of **PNBN** before and after photocatalysis.

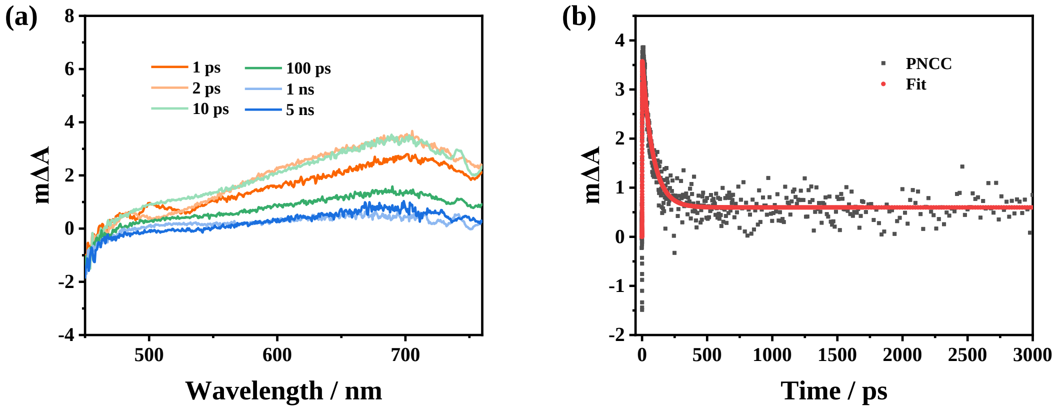

Figure S38. (a) Femtosecond transient absorption spectra of **PNCC**. (b) The overall decay kinetics of **PNCC**.

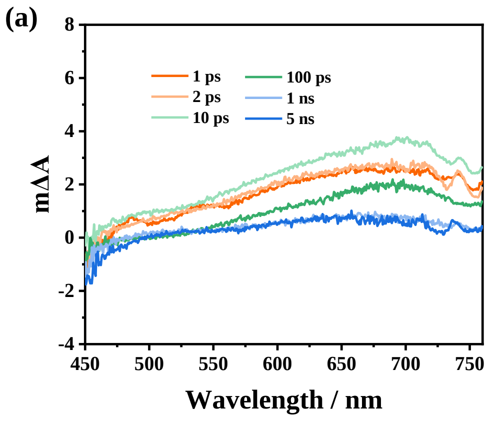

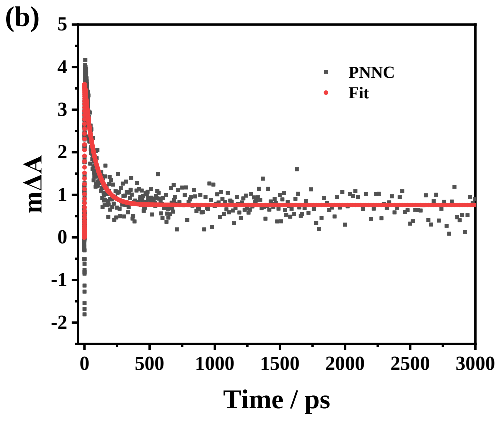

Figure S39. (a) Femtosecond transient absorption spectra of **PNNC**. (b) The overall decay kinetics of **PNNC**.

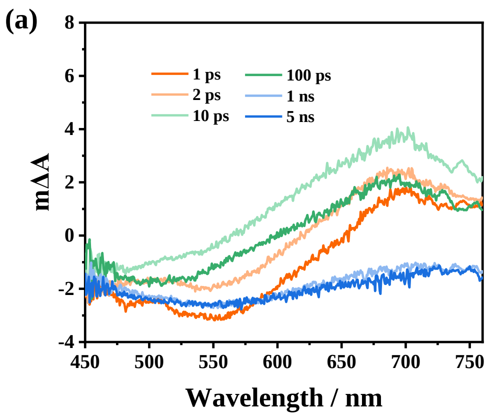

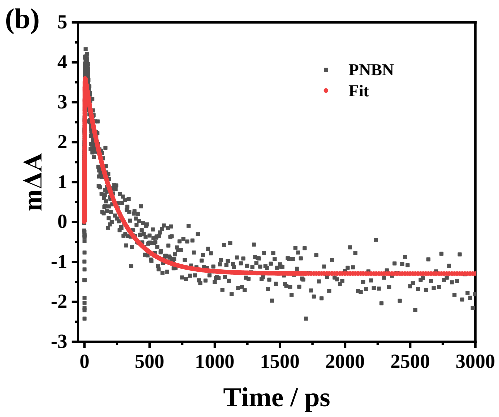

Figure S40. (a) Femtosecond transient absorption spectra of **PNBN**. (b) The overall decay kinetics of **PNBN**.

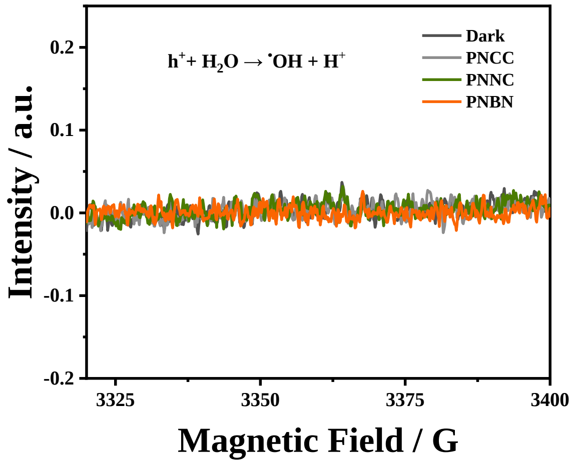

Figure S41. EPR spectra of polymers **PNCC**, **PNNC**, and **PNBN** for detection of ${}^{\bullet}$OH radicals in an aqueous dispersion.

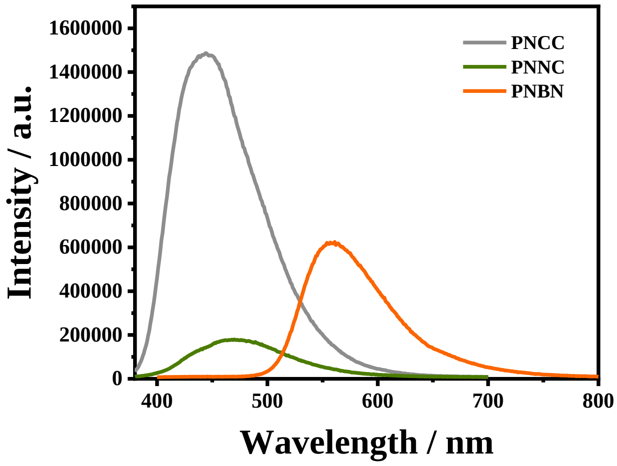

Figure S42. Fluorescence spectra of polymers **PNCC**, **PNNC**, and **PNBN** in solid.

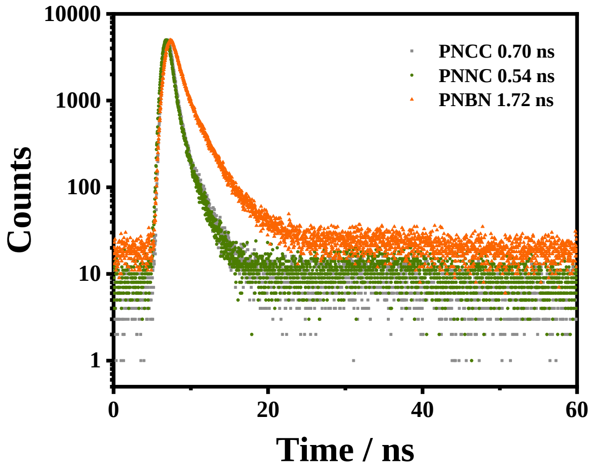

Figure S43. Photoluminescence decay traces of polymers **PNCC**, **PNNC**, and **PNBN** in the solid-state.

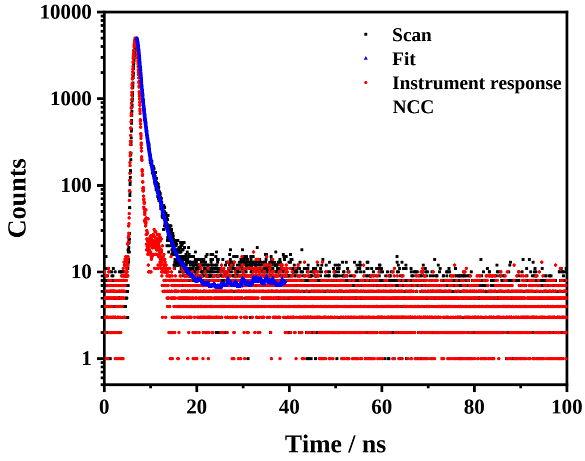

Figure S44. Photoluminescence decay traces of **PNCC** in the solid-state.

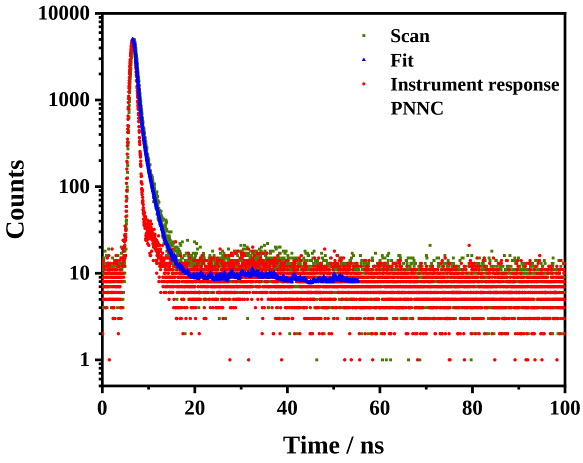

Figure S45. Photoluminescence decay traces of **PNNC** in the solid-state.

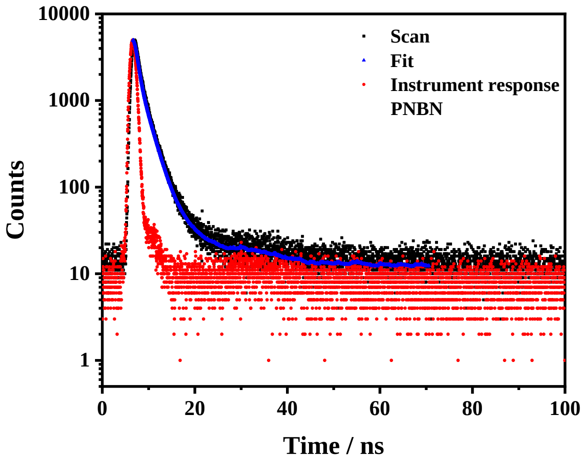

Figure S46. Photoluminescence decay traces of **PNBN** in the solid-state.

Table S6. Fluorescence lifetime properties of the polymers in the solid state.

Material	Emission (nm)	τ_1 (ns)	α_1 (%)	τ_2 (ns)	α_2 (%)	τ_3 (ns)	α_3 (%)	χ^2	τ_{AVG} (ns)
**PNCC**[a]	446	0.26	52.18	0.87	38.28	2.43	9.54	1.15	0.70
**PNNC**[a]	474	0.17	62.44	0.94	31.63	2.32	5.93	1.25	0.54
**PNBN**[a]	569	0.54	39.95	2.04	54.86	7.55	5.19	1.08	1.72

[a]Excitation wavelength λ_{ex} = 360 nm.

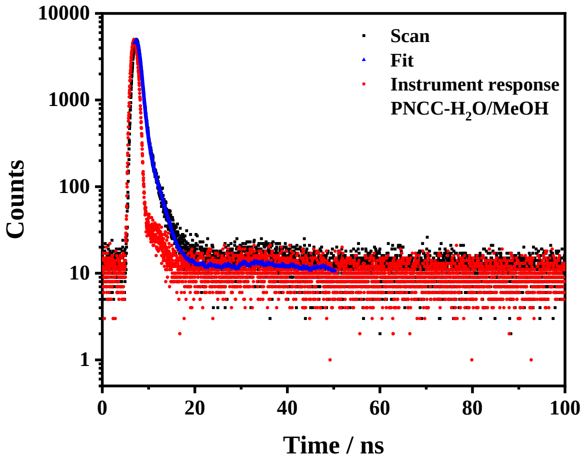

Figure S47. Photoluminescence decay traces of **PNCC** in H_2O/MeOH (1:1, 0.002 M) suspension.

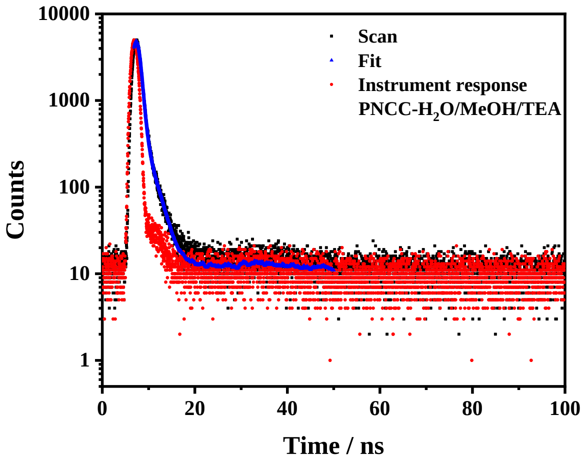

Figure S48. Photoluminescence decay traces of **PNCC** in H_2O/MeOH/TEA (1:1:1, 0.002 M) suspension.

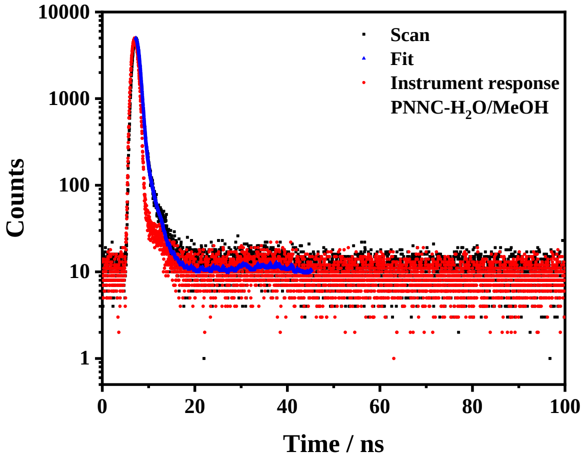

Figure S49. Photoluminescence decay traces of **PNNC** in H_2O/MeOH (1:1, 0.002 M) suspension.

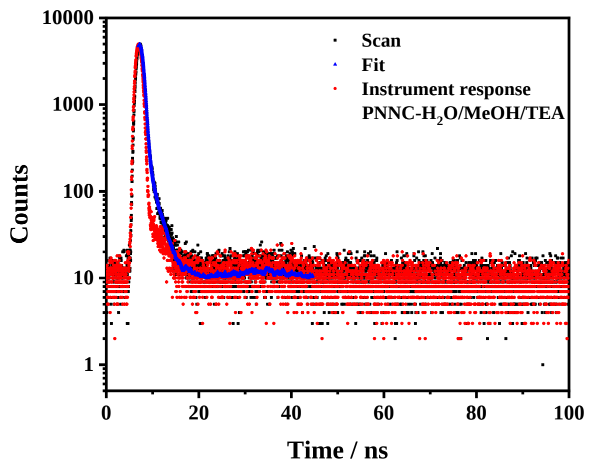

Figure S50. Photoluminescence decay traces of **PNNC** in H_2O/MeOH/TEA (1:1:1, 0.002M) suspension.

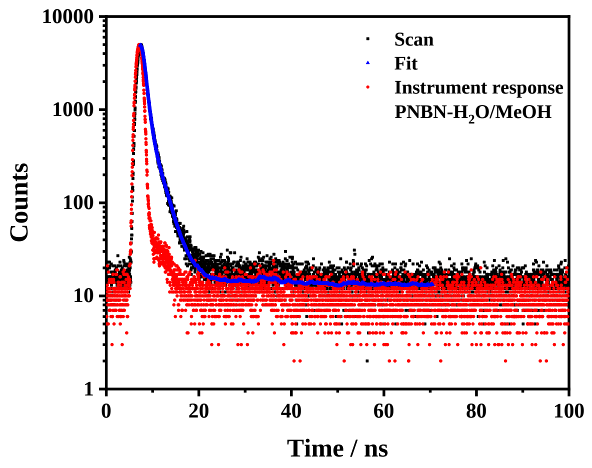

Figure S51. Photoluminescence decay traces of **PNBN** in H_2O/MeOH (1:1, 0.002 M) suspension.

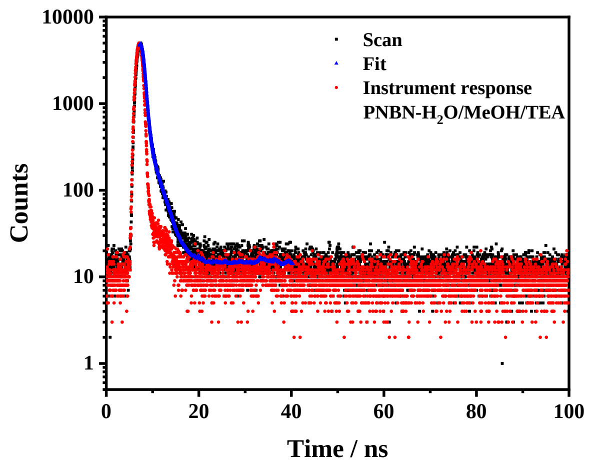

Figure S52. Photoluminescence decay traces of **PNBN** in H_2O/MeOH/TEA (1:1:1, 0.002 M) suspension.

Table S7. Fluorescence lifetime properties of the polymers in H_2O/MeOH or H_2O/MeOH/TEA suspension.

Material	Emission (nm)	τ_1 (ns)	α_1 (%)	τ_2 (ns)	α_1 (%)	χ^2	τ_{Av} (ns)
**PNCC**[a]	445	0.51	81.11	1.85	18.89	1.27	0.76
**PNCC**[b]	438	0.49	81.83	1.84	18.17	1.16	0.74
**PNNC**[a]	470	0.33	84.97	1.26	15.03	1.22	0.47
**PNNC**[b]	466	0.31	89.62	1.44	10.38	1.10	0.43
**PNBN**[a]	550	0.62	69.76	2.15	30.24	1.15	1.08
**PNBN**[b]	540	0.40	82.03	1.95	17.97	1.05	0.68

[a]Photoluminescence decay traces of polymers in H_2O/MeOH (1:1, 0.002 M) suspension. [b]Photoluminescence decay traces of polymers in H_2O/MeOH/TEA (1:1:1, 0.002 M) suspension.

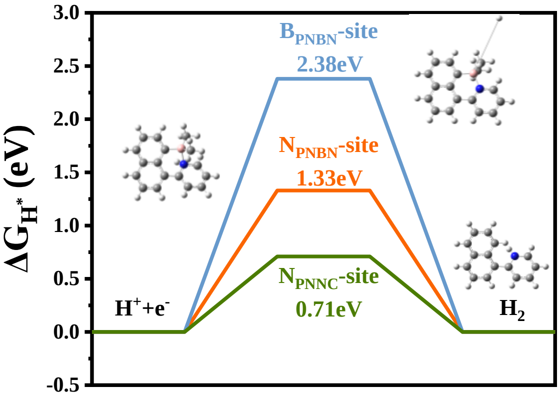

Figure S53. Hydrogen binding free energy (ΔG_H) at boron and nitrogen sites. The insets indicate the structures of transition states.

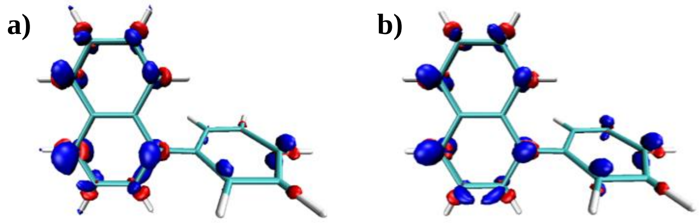

Figure S54. Isosurfaces of the charge density difference between the hole/electron polaron and ground state of the polymers fragment structure of **MNCC1-DFT**.

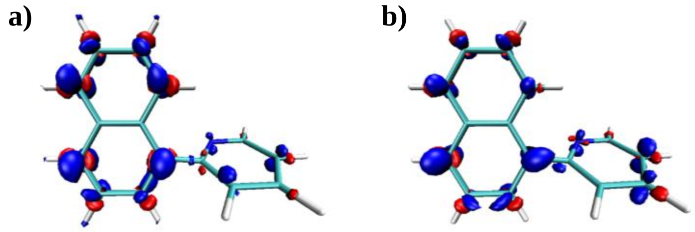

Figure S55. Isosurfaces of the charge density difference between the hole/electron polaron and ground state of the polymers fragment structure of **MNNC1-DFT**.

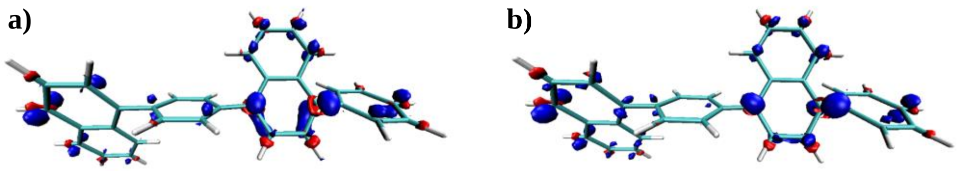

Figure S56. Isosurfaces of the charge density difference between the hole/electron polaron and ground state of the polymers fragment structure of **MNCC2-DFT**.

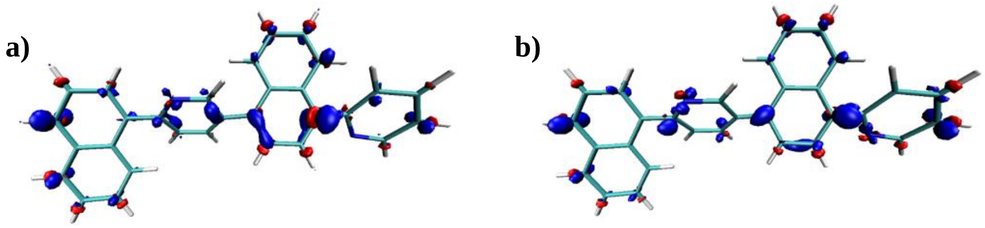

Figure S57. Isosurfaces of the charge density difference between the hole/electron polaron and ground state of the polymers fragment structure of **MNNC2-DFT**.

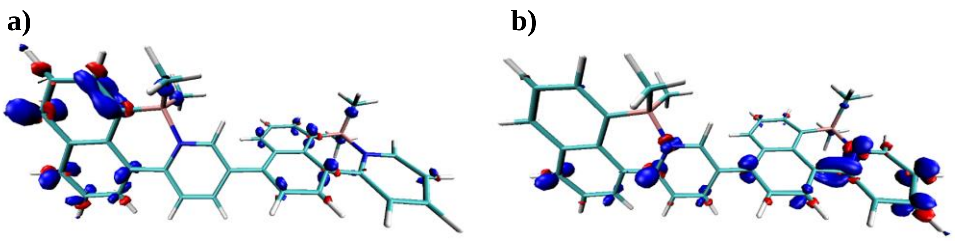

Figure S58. Isosurfaces of the charge density difference between the hole/electron polaron and ground state of the polymers fragment structure of **MNBN2-DFT**.

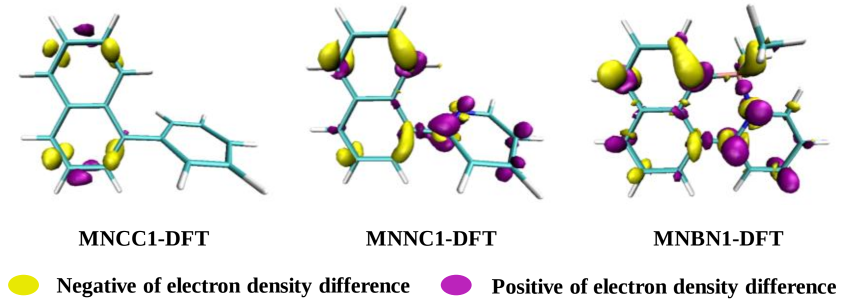

Figure S59. Electron density differences between the first excited state and the ground state in the fragments.

Table S8. HERs of reported conjugated polymers under visible light irradiation.

Polymers	Photocatalytic Conditions	HER (μmol h^{-1})	AQY	Reference
**PNBN**	10 mg polymer, 50 mL H_2O /CH_3OH /Et_3N (1/1/1), λ > 420 nm.	217.4	10.9 % (420 nm)	This work
**B-BT-1,4**	50 mg polymer, 110 mL TEOA/H_2O (1/10), 3 wt % Pt, λ > 420 nm.	116.0	4.01 % (420 nm)	*Angew. Chem., Int. Ed.* **2016**, *55*, 9202–9206.
**P7**	25 mg polymer, 22.5 mL H_2O /CH_3OH /Et_3N (1/1/1), λ > 420 nm.	92.0	7.2 % (420 nm)	*Angew. Chem., Int. Ed.* **2016**, *55*, 1792–1796.
**Flu-SO**	10 mg polymer, 50 mL H_2O /CH_3OH /Et_3N (3/1/1), λ > 420 nm.	50.4	2.13 % (420 nm)	*Small* **2018**, *14*, 1801839.
**P10**	25 mg polymer, 22.5 mL H_2O /CH_3OH /Et_3N (1/1/1), λ > 420 nm.	81.5	11.6 % (420 nm)	*Nat. Commun.* **2018**, *9*, 4968.
**P28**	25 mg polymer, 25 mL H_2O /CH_3OH /Et_3N (1/1/1), λ > 420 nm.	24.0	6.7 % (420 nm)	*Chem. Mater.* **2018**, *30*, 5733−5742.
**P10-e**	0.1 mg mL^{-1} polymer, 25 mL H_2O /CH_3OH /Et_3N (1/1/1) (aqueous phase containing water : toluene 9 : 1, SDS surfactant 10 mg mL^{-1} and Na_2CO_3 3.5 mg mL^{-1}) , λ > 420 nm.	36.3	5.8 % (420 nm)	*J. Mater. Chem. A* **2019**, *7*, 2490–2496.
**FSO-FS**	50 mg polymer, 110 mL TEOA/H_2O (1/10), λ > 420 nm.	170.0	6.8 % (420 nm)	*Angew. Chem., Int. Ed.* **2019**, *58*, 10236–10240.
**P-FSO**	50 mg polymer, 110 mL TEOA/H_2O (1/10), λ > 420 nm.	400.0	8.5 % (420 nm)	*Appl. Catal., B* **2019**, *245*, 596–603.
**FS-TEG**	25 mg polymer, 22.5 mL H_2O /CH_3OH /Et_3N (1/1/1), λ > 420 nm.	72.5	10.0 % (420 nm)	*Energy Environ. Sci.* **2020**, *13*, 1843–1855.
**PyDTDO-3**	10 mg polymer, 100 mL 1.0 M aqueous solution of AA containing 10 vol% DMF, λ > 420 nm.	163.2	3.7 % (420 nm)	*Chem. Sci.* **2021**, *12*, 1796–1802.
**8**	Colloidal platinum particles (Pt-PVP) as catalysts, and EDTA as electron sacrifice agent, the reaction was taken place in a pyrex bottle (20 mL) with buffer solution (pH = 5.0, 5 mL) under Xenon light (λ > 400 nm) at 100 mW	4.8	0.79 % (419 nm)	*Adv. Sci.* **2022**, *9*, 2101652

Cartesian coordinates for the optimized geometries

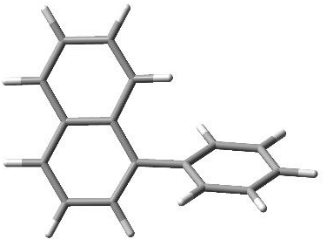

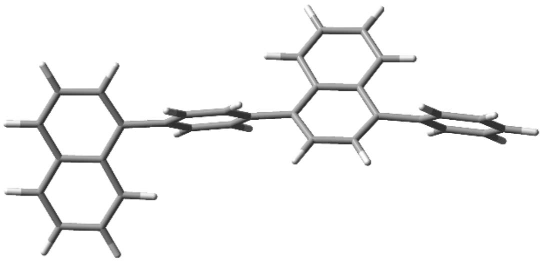

The fragmental structure of polymer **MNCC1-DFT**					The fragmental structure of polymer **MNCC2-DFT**				
Tag	Symbol	X	Y	Z	Tag	Symbol	X	Y	Z
1	C	0.0740092	0.7390400	0.0621167	1	C	4.1119237	-0.4438262	0.2580264
2	C	1.4801828	0.2436744	0.0314521	2	C	5.5652805	-0.7641691	0.1736382
3	C	2.3696724	0.6999211	-0.9502396	3	C	6.3054528	-1.0129726	1.3369878
4	C	-0.1536902	2.0958735	0.1907220	4	C	3.2610686	-1.3471244	0.8612796
5	C	-1.4600708	2.6254449	0.2576596	5	C	1.8719227	-1.1282465	0.9176185
6	C	-2.5466339	1.7908420	0.1970092	6	C	1.2944819	0.0001427	0.3722482
7	C	-2.3722351	0.3914346	0.0464530	7	C	2.1457952	0.9957660	-0.2123055
8	C	-1.0472653	-0.1513222	-0.0327594	8	C	3.5642179	0.7717269	-0.2705524
9	C	1.9606014	-0.6452779	1.0036540	9	C	6.2140249	-0.8753836	-1.0645926
10	C	3.2883683	-1.0634609	0.9930268	10	C	7.5601270	-1.2224395	-1.1355863
11	C	4.1615445	-0.6043412	0.0084542	11	C	8.2856351	-1.4629378	0.0299242
12	C	3.6973461	0.2788863	-0.9631237	12	C	7.6533388	-1.3573736	1.2662934
13	C	-3.4858098	-0.4846666	-0.0426965	13	C	1.6362861	2.2313406	-0.6969206
14	C	-3.3131626	-1.8346021	-0.2203284	14	C	2.4649907	3.1913847	-1.2239569
15	C	-2.0100141	-2.3696089	-0.3271239	15	C	3.8565996	2.9710287	-1.2822884
16	C	-0.9094750	-1.5513800	-0.2375822	16	C	4.3888465	1.7957241	-0.8116603
17	H	2.0103260	1.3800191	-1.7145302	17	C	-0.1881038	0.1414084	0.4045834
18	H	0.6951739	2.7659290	0.2682505	18	C	-0.8825592	0.1010469	1.6195093
19	H	-1.5961357	3.6953097	0.3692511	19	C	-2.2711838	0.1876890	1.6563111
20	H	-3.5541656	2.1887369	0.2577842	20	C	-3.0194232	0.3105887	0.4794545
21	H	1.2913687	-0.9950718	1.7816129	21	C	-2.3243667	0.3465361	-0.7379814
22	H	3.6429010	-1.7440171	1.7593346	22	C	-0.9372077	0.2665119	-0.7742772
23	H	5.1950694	-0.9317253	-0.0000333	23	C	-4.5018547	0.4519864	0.5346898
24	H	4.3679643	0.6393008	-1.7353888	24	C	-5.0460717	1.4982684	1.2554185
25	H	-4.4832073	-0.0626640	0.0255556	25	C	-6.4398543	1.7083937	1.3203969
26	H	-4.1731788	-2.4912640	-0.2889877	26	C	-7.2960011	0.8664478	0.6577086
27	H	-1.8785300	-3.4338979	-0.4876222	27	C	-6.7941008	-0.2347579	-0.0817188
28	H	0.0822076	-1.9733979	-0.3353912	28	C	-5.3792504	-0.4602885	-0.1418965
					29	C	-7.6673839	-1.1300239	-0.7536344
					30	C	-7.1800927	-2.2130638	-1.4415409
					31	C	-5.7889623	-2.4558035	-1.4780583
					32	C	-4.9146004	-1.6051713	-0.8450399
					33	H	5.8202471	-0.9218172	2.3024061

34	H	3.6660661	-2.2692621	1.2627973
35	H	1.2365518	-1.8867617	1.3610203
36	H	5.6526190	-0.7042560	-1.9761176
37	H	8.0413130	-1.3114239	-2.1033493
38	H	9.3344338	-1.7318279	-0.0258240
39	H	8.2098867	-1.5397490	2.1789777
40	H	0.5731684	2.4204442	-0.6288523
41	H	2.0502844	4.1276265	-1.5805567
42	H	4.5091624	3.7383527	-1.6835993
43	H	5.4602309	1.6462438	-0.8325299
44	H	-0.3262400	0.0104188	2.5458651
45	H	-2.7855873	0.1506272	2.6101732
46	H	-2.8770260	0.4509359	-1.6648121
47	H	-0.4260535	0.2913502	-1.7300498
48	H	-4.3814314	2.1902248	1.7604320
49	H	-6.8260283	2.5478795	1.8875450
50	H	-8.3683336	1.0274096	0.6932142
51	H	-8.7354326	-0.9441161	-0.7068734
52	H	-7.8592023	-2.8893205	-1.9484845
53	H	-5.4095772	-3.3245620	-2.0043785
54	H	-3.8529036	-1.8128700	-0.8694714

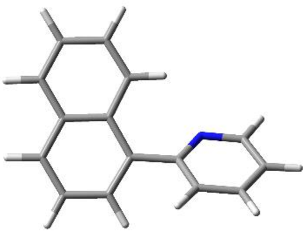

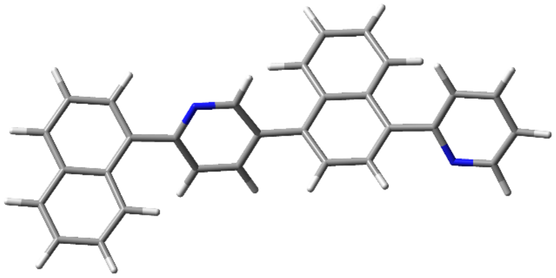

The fragmental structure of polymer **MNNC1-DFT**<br>Tag Symbol X Y Z					The fragmental structure of polymer **MNNC2-DFT**<br>Tag Symbol X Y Z				
Row	Symbol	X	Y	Z	Row	Symbol	X	Y	Z
1	C	-0.1031798	0.7079391	-0.0757066	1	C	-4.4101309	0.5726192	0.1757054
2	C	-1.5060428	0.2038073	-0.0277549	2	C	-2.9434768	0.3327193	0.0663450
3	N	-1.8286501	-0.8574611	-0.7858084	3	N	-2.1600988	0.9851906	0.9424162
4	C	0.0913593	2.0706725	-0.2104602	4	C	-4.8335340	1.8762331	0.3534960
5	C	1.3809221	2.6372752	-0.2707130	5	C	-6.2012373	2.2115955	0.3828146
6	C	2.4874265	1.8324588	-0.1859256	6	C	-7.1536343	1.2340062	0.2402118
7	C	2.3493082	0.4289416	-0.0362346	7	C	-6.7762017	-0.1252605	0.1010361
8	C	1.0407755	-0.1566811	0.0153117	8	C	-5.3849720	-0.4766258	0.0840527
9	C	-2.4609309	0.8531283	0.7710929	9	C	-2.3905261	-0.4609388	-0.9483383
10	C	-3.7763898	0.4074631	0.7579841	10	C	-1.0114448	-0.5912008	-1.0370907
11	C	-4.1104711	-0.6791685	-0.0442477	11	C	-0.1882699	0.0715303	-0.1209428

12	C	-3.0957337	-1.2758410	-0.7873903	12	C	-0.8394996	0.8501604	0.8468951
13	C	3.4915739	-0.4073494	0.0714792	13	C	-7.7539719	-1.1502409	0.0035426
14	C	3.3625426	-1.7633940	0.2339461	14	C	-7.3923979	-2.4713225	-0.0761654
15	C	2.0760774	-2.3427275	0.2961150	15	C	-6.0257814	-2.8263977	-0.0449571
16	C	0.9486881	-1.5643307	0.1890148	16	C	-5.0529384	-1.8587937	0.0338228
17	H	-0.7712149	2.7207480	-0.3027203	17	C	4.1030567	-0.5194020	-0.0369282
18	H	1.4879586	3.7092951	-0.3914785	18	C	5.5498981	-0.8382831	0.1291705
19	H	3.4849367	2.2568548	-0.2316993	19	N	6.0098577	-1.8914693	-0.5671964
20	H	-2.1650544	1.6821989	1.4015725	20	C	3.2105891	-1.5716758	-0.0139057
21	H	-4.5260933	0.8956748	1.3708291	21	C	1.8234140	-1.3589785	-0.0614983
22	H	-5.1230049	-1.0618620	-0.0882780	22	C	1.2891995	-0.0879831	-0.1440363
23	H	-3.3092238	-2.1341113	-1.4192874	23	C	2.1800582	1.0300883	-0.2551733
24	H	4.4741324	0.0509652	0.0271878	24	C	3.6012792	0.8133108	-0.2062435
25	H	4.2428412	-2.3910959	0.3160697	25	C	6.3674337	-0.1372381	1.0288964
26	H	1.9770934	-3.4146854	0.4266167	26	C	7.6941680	-0.5210177	1.1807518
27	H	-0.0273737	-2.0249153	0.2127802	27	C	8.1741183	-1.5958864	0.4388711
					28	C	7.2855889	-2.2484907	-0.4114515
					29	C	1.7019013	2.3510195	-0.4730811
					30	C	2.5617494	3.4094651	-0.6313327
					31	C	3.9548565	3.1961998	-0.6003752
					32	C	4.4560992	1.9333454	-0.4003355
					33	H	-4.0848154	2.6514900	0.4577417
					34	H	-6.4937287	3.2481950	0.5063337
					35	H	-8.2092363	1.4847262	0.2458346
					36	H	-3.0327995	-0.9424698	-1.6746156
					37	H	-0.5685709	-1.1865714	-1.8280233
					38	H	-0.2544988	1.3820337	1.5924682
					39	H	-8.8013448	-0.8666065	0.0061441
					40	H	-8.1500719	-3.2436141	-0.1454665
					41	H	-5.7424008	-3.8726776	-0.0728019
					42	H	-4.0142522	-2.1566521	0.0845642
					43	H	3.5977060	-2.5790969	0.0721009
					44	H	1.1569562	-2.2108932	0.0140670
					45	H	5.9590050	0.6768939	1.6137860
					46	H	8.3391739	0.0048635	1.8759332
					47	H	9.2017321	-1.9285127	0.5222072
					48	H	7.6128686	-3.1028175	-0.9982614
					49	H	0.6348899	2.5168224	-0.5393360
					50	H	2.1696719	4.4054026	-0.8034520
					51	H	4.6326883	4.0280238	-0.7558773
					52	H	5.5263559	1.7800709	-0.4224228

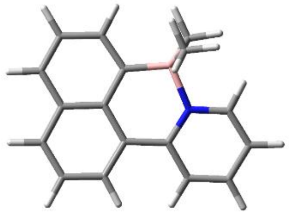

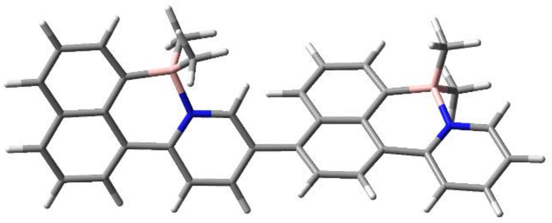

The fragmental structure of polymer **MNBN1-DFT**

Tag	Symbol	X	Y	Z
1	C	0.2294766	1.2409047	0.0252175
2	C	-1.2182288	0.9450806	-0.0183036
3	C	-2.1760848	1.9763247	-0.1139152
4	C	0.6529646	2.5627786	0.0994795
5	C	2.0139971	2.9080447	0.1004773
6	C	2.9701865	1.9268180	0.0235310
7	C	2.6025836	0.5611313	-0.0319960
8	C	1.2120403	0.1915566	-0.0160387
9	N	-1.6591755	-0.3393088	0.0234240
10	C	-2.9841469	-0.5985369	0.0190106
11	C	-3.9511299	0.3787452	-0.0449220
12	C	-3.5281700	1.7047041	-0.1241127
13	C	3.5914676	-0.4492716	-0.0906346
14	C	3.2207611	-1.7705342	-0.1203163
15	C	1.8580327	-2.1213344	-0.0852700
16	C	0.8379983	-1.1818990	-0.0314468
17	B	-0.6875319	-1.6811798	0.0611985
18	C	-0.9769544	-2.4235801	1.4876380
19	C	-1.0961509	-2.5909327	-1.2341117
20	H	-1.8507194	3.0011002	-0.1914645
21	H	-0.0600490	3.3716847	0.1691048
22	H	2.2978573	3.9523433	0.1596519
23	H	4.0243863	2.1843762	0.0140956
24	H	-3.2447659	-1.6448568	0.0653294
25	H	-4.9979388	0.1061261	-0.0440209
26	H	-4.2448762	2.5144118	-0.1968304
27	H	4.6368602	-0.1598640	-0.1061894
28	H	3.9756603	-2.5486916	-0.1634346
29	H	1.6008070	-3.1757085	-0.0933391
30	H	-2.0042005	-2.7974867	1.5920198
31	H	-0.3221377	-3.2966661	1.5863159
32	H	-0.7736203	-1.7800009	2.3521709
33	H	-1.0132792	-2.0434878	-2.1811243
34	H	-0.4164959	-3.4467906	-1.3097592
35	H	-2.1074565	-3.0154266	-1.1882038

The fragmental structure of polymer **MNBN2-DFT**

Tag	Symbol	X	Y	Z
1	C	-4.7841801	-1.0646470	-0.5657203
2	C	-3.3159667	-1.0574656	-0.4137052
3	C	-2.5257896	-2.1350250	-0.8579226
4	C	-5.3841792	-2.0551998	-1.3345875
5	C	-6.7794619	-2.1534954	-1.4584053
6	C	-7.5904384	-1.2607671	-0.8030442
7	C	-7.0365631	-0.2118466	-0.0303493
8	C	-5.6084944	-0.0801755	0.0795107
9	N	-2.6871026	-0.0085736	0.1787564
10	C	-1.3424680	0.0161491	0.2626406
11	C	-0.5128747	-0.9932897	-0.2003208
12	C	-1.1513400	-2.1085647	-0.7585791
13	C	-7.8730542	0.7238476	0.6233171
14	C	-7.3181034	1.7550442	1.3394952
15	C	-5.9186159	1.8916092	1.4136232
16	C	-5.0410497	1.0084586	0.8007689
17	B	-3.4585274	1.2980726	0.8528649
18	C	-2.9187844	1.4290335	2.3890762
19	C	-3.0699994	2.5823575	-0.0795361
20	C	3.7822192	-0.9381282	0.3612027
21	C	5.2446855	-1.0268847	0.5590627
22	C	5.8416440	-2.1924024	1.0813581
23	C	2.9703259	-1.9426939	0.8685935
24	C	1.5841371	-1.9390579	0.6751002
25	C	0.9631487	-0.9341564	-0.0395832
26	C	1.7533022	0.1260929	-0.5871911
27	C	3.1780855	0.1505605	-0.3554487
28	N	6.0482825	0.0158069	0.2276585
29	C	7.3753714	-0.0537353	0.4641312
30	C	7.9930593	-1.1561631	1.0108640
31	C	7.2001536	-2.2620966	1.3119939
32	C	1.1740036	1.1546089	-1.3699849
33	C	1.9504773	2.1808045	-1.8503002
34	C	3.3246372	2.2243668	-1.5634684
35	C	3.9673366	1.2367280	-0.8318012
36	B	5.5326947	1.4091846	-0.4983014

37	C	5.7679651	2.6099938	0.5839777
38	C	6.4218787	1.5865105	-1.8584656
39	H	-3.0007904	-3.0068554	-1.2790814
40	H	-4.7853907	-2.7757668	-1.8739057
41	H	-7.2054938	-2.9408519	-2.0693843
42	H	-8.6701981	-1.3384189	-0.8796244
43	H	-0.9305072	0.8894890	0.7447253
44	H	-0.5637142	-2.9448508	-1.1197355
45	H	-8.9491434	0.6135983	0.5410736
46	H	-7.9554006	2.4757531	1.8413168
47	H	-5.5161616	2.7349517	1.9654081
48	H	-3.4865098	2.2069476	2.9110404
49	H	-3.0525463	0.5064163	2.9675606
50	H	-1.8651465	1.7223917	2.4768082
51	H	-3.3858487	2.4626785	-1.1232383
52	H	-3.5722962	3.4798768	0.2987772
53	H	-1.9952469	2.8080491	-0.0846674
54	H	5.2311321	-3.0551855	1.2938796
55	H	3.3822504	-2.7499625	1.4569393
56	H	0.9888447	-2.7279337	1.1204073
57	H	7.9363376	0.8249830	0.1850194
58	H	9.0618055	-1.1506372	1.1776940
59	H	7.6376584	-3.1673630	1.7163159
60	H	0.1167713	1.1266449	-1.5963064
61	H	1.4984492	2.9646987	-2.4485153
62	H	3.9004530	3.0664949	-1.9324068
63	H	6.8234192	2.7587952	0.8481686
64	H	5.4194248	3.5596305	0.1630347
65	H	5.2152232	2.4586819	1.5189705
66	H	6.0445751	2.4366683	-2.4366519
67	H	7.4834092	1.7988339	-1.6789764
68	H	6.3691386	0.7131010	-2.5201809

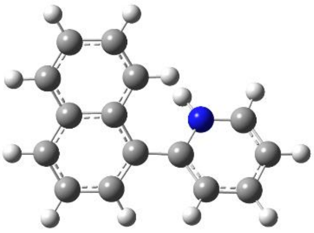

Tag	Symbol	X	Y	Z	Tag	Symbol	X	Y	Z
1	C	0.0849900	0.7517790	0.0144850	15	C	-2.0654470	-2.3397340	-0.3790290
2	C	1.4693950	0.2891020	-0.0171460	16	C	-0.9528850	-1.5311220	-0.3145040
3	N	1.8179160	-0.8966620	0.6651170	17	H	0.6901710	2.7938030	0.2242190
4	C	-0.1548160	2.1211730	0.1390760	18	H	-1.5815680	3.7198470	0.3320290
5	C	-1.4516740	2.6484550	0.2257180	19	H	-3.5497480	2.2142000	0.3178260

6	C	-2.5460730	1.8149450	0.2213450	20	H	2.2447550	1.8446760	-1.2337790
7	C	-2.3752050	0.4147390	0.0791570	21	H	4.5898880	1.0271410	-1.1942670
8	C	-1.0561320	-0.1376540	-0.0529770	22	H	5.1256660	-1.0894910	0.1044850
9	C	2.5009860	0.9580760	-0.6671820	23	H	3.2813610	-2.2614990	1.2869020
10	C	3.8164640	0.5003130	-0.6521910	24	H	-4.4868230	-0.0193320	0.1478230
11	C	4.1182290	-0.6952270	0.0713000	25	H	-4.2241290	-2.4452110	-0.2292660
12	C	3.1258730	-1.3541070	0.7188740	26	H	-1.9495150	-3.3969430	-0.5904160
13	C	-3.4988230	-0.4518160	0.0276940	27	H	0.0201750	-1.9662660	-0.4974080
14	C	-3.3537490	-1.8000190	-0.1875590	28	H	1.1475500	-1.2777030	1.3136610

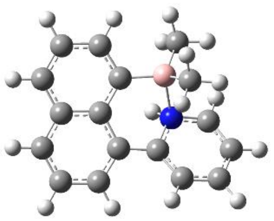

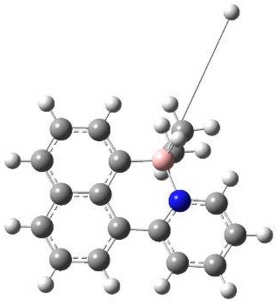

Tag	Symbol	X	Y	Z	Tag	Symbol	X	Y	Z
Row	Symbol	X	Y	Z	Row	Symbol	X	Y	Z
1	C	-0.3180829	-1.2461165	-0.0596423	1	C	0.3149827	-1.2816373	0.0261852
2	C	1.1194865	-1.0154049	-0.0667662	2	C	-1.1462522	-1.0676125	-0.0259509
3	C	2.0675329	-1.8472432	0.4864888	3	C	-2.0416812	-2.1501784	-0.1520289
4	C	-0.7998280	-2.5503708	-0.0964138	4	C	0.8111850	-2.5773662	0.1092767
5	C	-2.1772866	-2.8208452	-0.1130946	5	C	2.1893461	-2.8463473	0.1060341
6	C	-3.0813977	-1.7855135	-0.1095849	6	C	3.0888020	-1.8139139	0.0146924
7	C	-2.6445950	-0.4372425	-0.0714149	7	C	2.6453829	-0.4710607	-0.0452162
8	C	-1.2408776	-0.1388804	-0.0388849	8	C	1.2366660	-0.1791361	-0.0198815
9	N	1.5454120	0.2336386	-0.6777964	9	N	-1.6601440	0.1885591	0.0338190
10	C	2.9648047	0.3992609	-0.8807713	10	C	-2.9976145	0.3711719	0.0274337
11	C	3.8647541	-0.4519010	-0.3400026	11	C	-3.9062341	-0.6592245	-0.0589488
12	C	3.4403625	-1.5931632	0.3892831	12	C	-3.4071977	-1.9565996	-0.1642119
13	C	-3.5797133	0.6270295	-0.0382716	13	C	3.5755747	0.5930423	-0.1143942
14	C	-3.1436847	1.9250490	0.0361836	14	C	3.1310779	1.8913090	-0.1421069
15	C	-1.7624287	2.2079687	0.0834840	15	C	1.7511859	2.1659469	-0.0926579
16	C	-0.7914023	1.2192293	0.0544916	16	C	0.7857903	1.1710414	-0.0289523
17	B	0.7660541	1.6275324	0.2061001	17	B	-0.7644242	1.5822377	0.0965495
18	C	1.3252838	1.5800747	1.7144986	18	C	-1.2426916	2.5054016	-1.1646649
19	C	1.2088995	2.9290166	-0.6435180	19	C	-1.0739366	2.2649629	1.5484671
20	H	1.7179080	-2.7179260	1.0264808	20	H	-1.6556456	-3.1516000	-0.2542069
21	H	-0.0999793	-3.3755608	-0.1535384	21	H	0.1432494	-3.4230168	0.1905828
22	H	-2.5196044	-3.8488173	-0.1493206	22	H	2.5313939	-3.8726156	0.1723458
23	H	-4.1474548	-1.9862058	-0.1344595	23	H	4.1556791	-2.0121130	-0.0009302
24	H	3.2282656	1.2806937	-1.4438900	24	H	-3.3179458	1.3999022	0.0897034

25	H	4.9193429	-0.2472116	-0.4800165	25	H	-4.9670589	-0.4476753	-0.0577466
26	H	4.1600245	-2.2566905	0.8485182	26	H	-4.0757815	-2.8041824	-0.2600551
27	H	-4.6391652	0.3946533	-0.0651831	27	H	4.6354063	0.3628650	-0.1384011
28	H	-3.8588697	2.7405122	0.0629274	28	H	3.8408249	2.7103991	-0.1932640
29	H	-1.4576922	3.2467497	0.1516797	29	H	1.4354223	3.2042451	-0.0956181
30	H	2.4128669	1.7077075	1.7710027	30	H	-2.2701315	2.8836978	-1.0864126
31	H	0.8835434	2.4129819	2.2750659	31	H	-0.6035155	3.3924321	-1.2317205
32	H	1.0649948	0.6679448	2.2613223	32	H	-1.1564475	1.9868766	-2.1275235
33	H	0.9752033	2.8730784	-1.7156718	33	H	-0.8129034	1.6129972	2.3908893
34	H	0.6705483	3.8059860	-0.2670338	34	H	-0.4762356	3.1765649	1.6606263
35	H	2.2724962	3.1713103	-0.5416383	35	H	-2.1219022	2.5659921	1.6798109
36	H	1.0584544	0.3519762	-1.5685583	36	H	-2.1275931	7.4253315	-0.3960106
